# Electron-Beam-Conditioned Magnetic-Dipole Emission in Cathodoluminescence

**DOI:** 10.1021/acsnanoscienceau.6c00074

**Published:** 2026-07-07

**Authors:** Gour Mohan Das, Kameyab Raza Abidi, Govindan Vadivel

**Affiliations:** † Nanoscience Center, Department of Chemistry, 205518 University of Jyväskylä, Jyväskylä 40014, Finland; ‡ Nanoscience Center, Department of Physics, University of Jyväskylä, Jyväskylä 40014, Finland; § Functional Material Technology Group, 656563 Ensemble^3^Centre of Excellence, Wólczyńska 133, Warsaw 01-919, Poland

**Keywords:** cathodoluminescence, magnetic-dipole, core−shell nanophotonics, aloof electron beam, anapole, Mie resonances

## Abstract

Accessing the magnetic-dipole (MD) optical response in nanoscale structures is a longstanding goal in photonics because magnetic modes enable low-loss and interference-rich control of light. In practice, MD signatures are often mixed with electric dipole and higher-order radiation, making selective radiative emission difficult. Here we show that aloof fast-electron cathodoluminescence (CL) provides an electron-beam-conditioned route to MD-dominated emission from core–shell nanoparticles. Through multipolar CL analysis, guided synthetic index design, and real-material refinement, we identify conditions under which the MD becomes the dominant radiative channel in CL. The beam-conditioned optimum differs from the conventional optical plane-wave benchmark, showing that electron-beam excitation can access nanoparticle mode structure in a distinct way. These results connect ideal magnetic-dipole scattering concepts to aloof-electron cathodoluminescence and provide a design route for identifying magnetic-dipole-favored radiative emission from core–shell Mie resonators.

## Graphical abstract

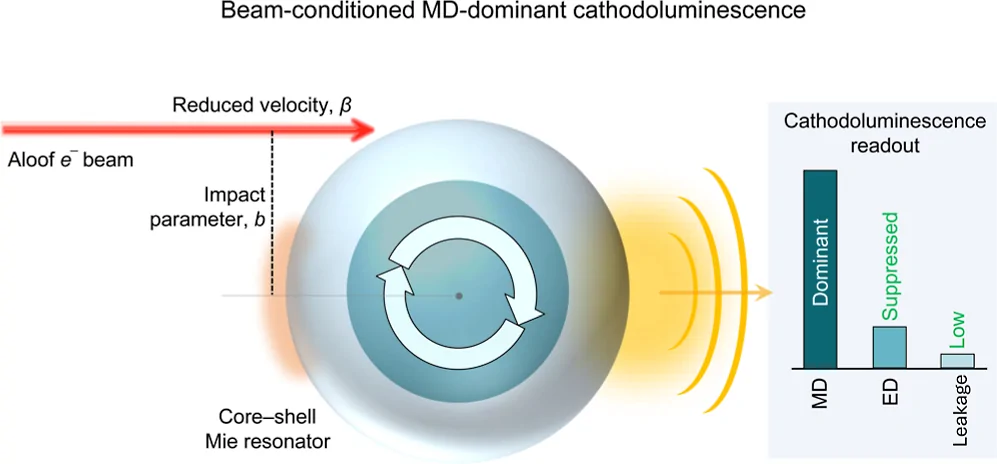

## Introduction

Magnetic-dipole response has become an important theme in nanophotonics because it opens a route to low-loss and interference-rich control of light at the nanoscale.^1–4^ In high-index dielectric nanostructures, optical Mie resonances can generate pronounced magnetic responses together with strong field localization, making them attractive for nanoantennas, directional scattering, metasurfaces, and other resonant photonic functionalities.^5–10^ In dielectric nanoparticles, MD modes typically coexist with electric dipole (ED) and higher-order multipoles, so the measured spectrum is often a mixed response rather than a clean magnetic one. This makes selective identification of MD emission a practical challenge in radiative mode assignment.^3,6,11^ Such identification does not conflict with the use of modal interference in nanophotonic design; interference among electric, magnetic, and higher-order channels remains essential for directional scattering, nanoantenna function, and resonant light control. A mode-resolved magnetic reference is useful for assigning CL spectral features to specific radiative multipoles, benchmarking fast-electron coupling to Mie resonances, and identifying conditions where MD emission dominates the recorded signal.

A useful benchmark for this problem is the ideal-magnetic-dipole scattering concept developed in plane-wave scattering, where the ED channel is suppressed while the MD remains resonant and HO multipoles stay weak.^12,13^ In this framework, ED suppression is closely linked to the anapole condition, arising from destructive interference between the Cartesian ED and the toroidal dipole (TD) so that the cleanest magnetic response is obtained when the MD resonance overlaps spectrally with an anapole-assisted ED minimum. Core–shell nanoparticles are especially attractive in this context because they offer substantially greater tuning freedom than homogeneous spheres: by varying the core radius, shell thickness, and optical contrast, one can reshape the spectral positions and line widths of the relevant multipolar channels and thereby create a broader design space for approaching MD-favored operation. Recent high-throughput screening of core–shell nanoparticles has further shown that ideal magnetic multipole conditions occupy restricted regions of material and geometry space, highlighting the need for guided selection of core–shell composition, size, and modal operating wavelength.^14^

At the same time, the measured response of a nanophotonic system is not determined by its intrinsic modal structure alone. It also depends on the excitation and probing schemes through which that structure is accessed. This point becomes especially important when one moves beyond plane-wave illumination to localized charged particle excitation. A fast electron beam does not probe the nanoparticle through the same spatial symmetry as a transverse optical wave; instead, it couples to the multipoles through trajectory-dependent weights, so the operational visibility of electric- and magnetic-type modes can change even when the underlying particle modes remain the same.^15,16^

Fast-electron spectroscopy offers a particularly powerful way to interrogate optical modes at the nanoscale because a swift electron carries a highly localized evanescent electromagnetic field that can couple to excitations beyond the reach of conventional light-based probes.^17^ In nanophotonic systems, this localized interaction has made electron-beam techniques especially valuable for accessing dark, higher-order, and strongly confined modes, while CL provides direct information on the radiative part of the response.^15–20^ For the present problem, this distinction is crucial: the goal is to determine whether a magnetic-type mode can become the dominant channel in a measurable emission signal, rather than merely confirming its existence in the nanoparticle. Plane-wave scattering nevertheless remains the natural reference for defining the intrinsic optical benchmark of MD behavior in dielectric nanoparticles. In particular, plane-wave studies of ideal or near-ideal MD response have shown that MD enhancement can be linked to anapole-assisted suppression of the ED, thereby providing a clear benchmark for magnetic-mode engineering in core–shell systems. The remaining question is how that benchmark appears when the same particle is probed through a different excitation scheme. Recent theory for core–shell nanoparticles indicates that, in dielectric systems, the spectral visibility of magnetic-type modes can depend strongly on electron-beam trajectory and may even be suppressed or obscured under unfavorable conditions, whereas aloof excitation preserves that visibility more effectively.^20^ Controlled electron beams have already been used to excite, map, and directionally shape Mie-mode cathodoluminescence in dielectric nanoparticles; however, the translation of plane-wave ideal-magnetic-dipole criteria, including anapole-assisted ED suppression, into a beam-weighted aloof-electron CL design problem for core–shell resonators remains largely unexplored. This makes aloof-electron CL a natural framework for asking how an optically defined MD benchmark is re-expressed under beam-conditioned radiative readout.

In this work, we investigate electron-beam-conditioned MD response in CL from concentric core–shell nanoparticles, using plane-wave scattering only as the intrinsic optical benchmark for the underlying mode structure. We combine synthetic index design, multipolar CL analysis, and real-material refinement to determine whether an MD-favored optical benchmark can be re-expressed as a beam-conditioned radiative response under aloof-electron excitation. We identify a CL regime in which the MD becomes the dominant radiative channel and show that its operating point is governed by the interplay of beam trajectory, wavelength, and core–shell geometry. The electron beam optimum does not coincide trivially with the conventional optical benchmark, indicating that the observable magnetic response depends on both the intrinsic mode structure of the particle and the excitation pathway through which it is probed. These results identify a beam-conditioned route to magnetic-mode selection in nanoscale photonics and broaden the role of CLs as a nanophotonic probe. By linking an optically defined benchmark to an aloof-electron radiative readout and extending the analysis to real-material candidates, this work opens a route toward electron-beam-controlled magnetic nanophotonics.

### Theory and Framework

We consider a concentric core–shell sphere embedded in air, with core radius *r*, outer radius *R*, and complex refractive indices *m*_1_(λ) = *n*_1_(λ) + i*k*_1_(λ) and *m*_2_(λ) = *n*_2_(λ) + i*k*_2_(λ) for the core and shell, respectively. The host medium is air, and all media are taken as nonmagnetic. The free-space wavenumber is *k* = 2π/λ. Under plane-wave illumination, the optical response is described by the electric and magnetic Mie coefficients $a_{l}$ and $b_{l}$ , evaluated using standard core–shell Mie theory.^21,22^ All calculations were performed using in-house Python routines based on a semianalytical multipolar formulation, following the standard fast-electron optical excitation framework and related CL treatments for spherical nanostructures.^16,20,23^ By employing a mesh-free semianalytical model, we eliminate spatial discretization artifacts. In this context, our numerical analysis explicitly comprises wavelength scans, parameter-space optimizations, and finite multipole summations. Additional implementation details and convergence controls are provided in Section S1.1 of the Supporting Information. The total scattering is decomposed into ED, MD, electric-quadrupole (EQ), magnetic-quadrupole (MQ), and HO contributions, *Q*_tot_ = *Q*_ED_ + *Q*_MD_ + *Q*_EQ_ + *Q*_MQ_ + *Q*_HO_. The corresponding plane-wave MD purity is defined as

$$\eta_{MD}^{PW}=\frac{Q_{MD}}{Q_{tot}}$$

A large η_MD_^PW^ is useful, however, it is not sufficient to establish an ideal MD state because a strong magnetic resonance can reduce the relative ED contribution without making that channel intrinsically small.

To distinguish genuine ED suppression from simple magnetic overshadowing, we supplement the spherical multipole decomposition with a Cartesian dipole analysis. The induced current density yields the Cartesian ED *P* and TD *T*, and the anapole condition is monitored through *P* = −*ikT*. Under these conditions, the far-field radiation of the Cartesian electric and toroidal dipoles cancels, so the spherical ED channel is driven to zero while the internal field remains finite. In practice, we quantify this through the mismatch parameter:

$$\Delta_{PT}=\frac{\left|P+ikT\right|}{\left|P\right|+\left|ikT\right|}$$

so that small Δ_PT_ indicates anapole-assisted ED suppression. This Cartesian diagnostic follows the electric–toroidal multipole description commonly used to identify anapole-assisted electric-dipole suppression.^24^

For the electron-driven problem, the particle is excited by a fast electron traveling outside the nanoparticle on an aloof trajectory with impact parameter *b* > *R*, following the standard fast-electron optical excitation framework.^16^ For a spherical particle, the response can be written in terms of beam-weighted electric- and magnetic-type scattered-field coefficients, here denoted $T_{El}^{33}$ and $T_{Ml}^{33}$ .^20^ The radiative CL signal is decomposed as, Γ_CL_ = Γ_ED_^CL^ + Γ_MD_^CL^ + Γ_EQ_^CL^ + Γ_MQ_^CL^ + Γ_HO_^CL^. The corresponding cathodoluminescence MD purity is $\eta_{MD}^{CL}=\frac{\Gamma_{MD}^{CL}}{\Gamma_{CL}}$ . Importantly, the total CL signal does not have to vanish at the anapole; only the ED contribution is expected to become negligible, while MD and HO radiative channels may remain finite. A central conceptual point of this work is that the intrinsic anapole condition remains a structural property of the nanoparticle, whereas the operational CL optimum is beam-conditioned. We therefore distinguish the plane-wave MD wavelength, λ_IMD_^PW^ from the beam-conditioned CL wavelength λ_IMD_^CL^(b,v). Their difference reflects the different ways in which optical and electron-beam excitation reveal the same class of modes. Additional details on the semianalytical implementation, beam-weighted multipolar decomposition, numerical controls, selection metrics, and real-material parameters are given in Sections S1.1, S2, S5, and S6 of the Supporting Information. The aloof-electron excitation geometry, the core–shell resonator, and the principal beam–control parameters are summarized schematically in Figure 1.

**Figure 1:**
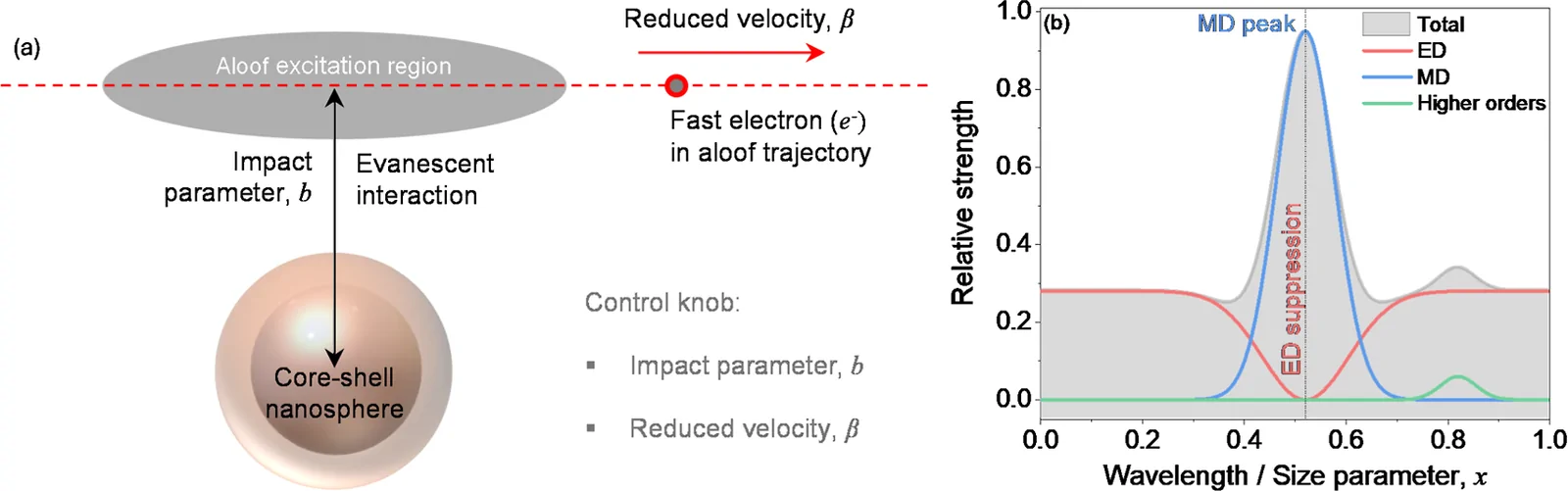
(a) Schematic of aloof fast-electron excitation of a concentric core–shell nanosphere. A fast electron travels with velocity *v* along a trajectory separated from the particle by the impact parameter *b*. The shaded region indicates the evanescent near-field interaction zone through which the electron excites the nanoparticle without direct penetration. The principal external control parameters are therefore the impact parameter *b* and the reduced electron velocity β = *v*/*c*. (b) Conceptual representation of the MD benchmark. At the selected wavelength, the MD contribution dominates, the ED contribution is strongly suppressed, and HO multipoles remain weak.

## Results

### Plane-Wave Branch: Candidate Selection, Anapole Validation, and Far-Field Limitation

We begin with the plane-wave excitation because it provides the most direct optical reference for identifying a MD-favored state in a core–shell nanosphere.^12^ In that case, the scattered response can be decomposed unambiguously into spherical multipolar channels, and the same candidate can then be examined from two complementary viewpoints: first through its multipolar spectrum, and second through the Cartesian electric- and toroidal-dipole picture. We first consider the lossless plane-wave problem to identify the intrinsic MD-favored state of the core–shell particle without the ambiguity introduced by dissipative broadening. This is essential because the purpose of the plane-wave branch is to determine whether the reduced ED response is genuinely linked to anapole-assisted *P*–*T* cancellation rather than to absorption-driven weakening of the spectrum. After the lossless benchmark is selected, we introduce a lossy counterpart of the same geometry only as a robustness test.

Figures 2 and S1 summarize the lossless plane-wave search in synthetic core–shell parameter space. The screening was performed over refractive-index and geometry combinations, and the selected candidate was identified from a composite score that favors strong MD response together with reduced ED, EQ, and MQ contributions. As shown in Figure S1a,b, the selected lossless candidate is located at *n*_core_ = 4.148, *n*_shell_ = 1.774, *r* = 124.2 nm, and *R* = 231.7 nm. The corresponding multipolar spectrum, plotted in Figure 2a, shows that the selected state occurs at λ_sel_^PW,0^ = 425.0 nm. At this wavelength, the multipolar response is strongly MD-favored, with *Q*_ED_ = 0.0010, *Q*_MD_ = 0.2214, *Q*_EQ_ = 0.0015, and *Q*_MQ_ = 0.0094. Figure 2b clarifies how this wavelength is selected by the screening metric: the score is not determined by MD strength alone; rather, it peaks only when the MD purity is high while the ED, EQ, and MQ suppression factors are simultaneously favorable. The sharp maximum near 425 nm therefore identifies a spectral point where MD response and non-MD-channel suppression coincide, rather than a point selected only from the largest MD amplitude.

**Figure 2:**
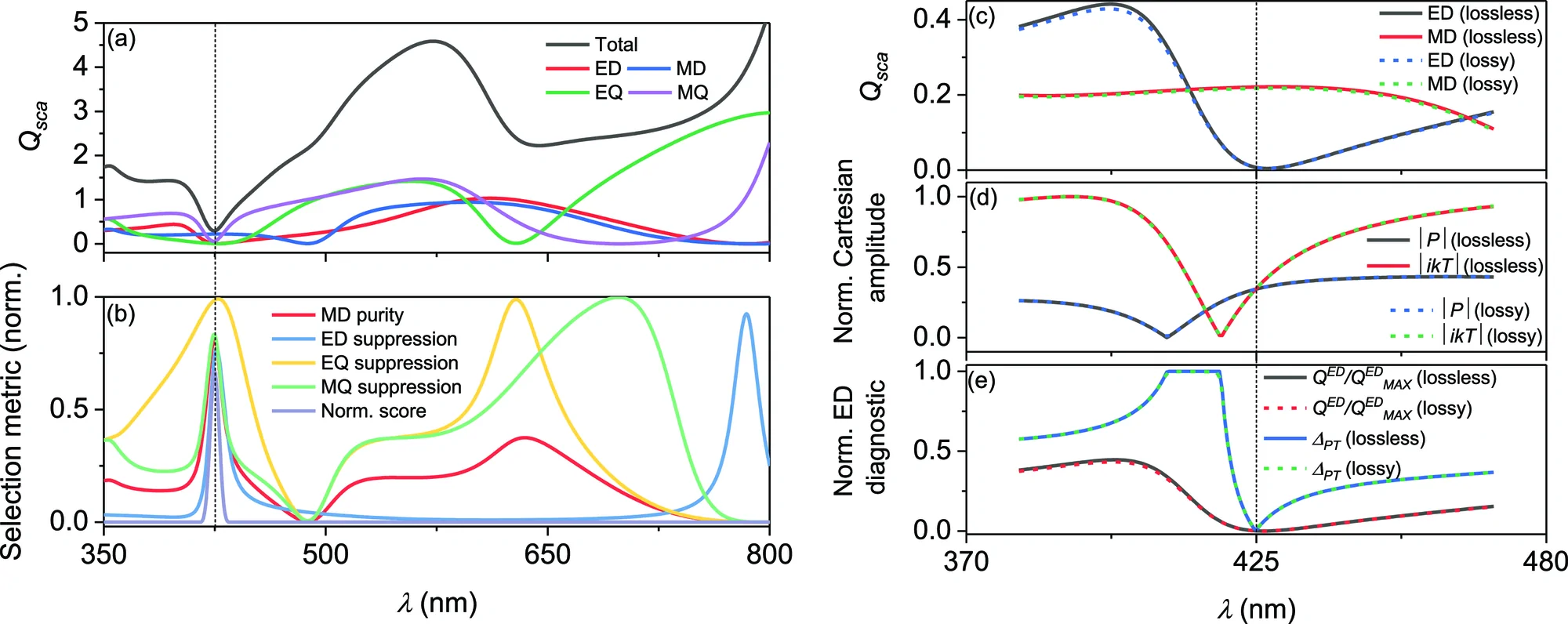
(a) Multipolar scattering spectrum of the selected candidate, showing the total *Q*_sca_ response together with ED, MD, EQ, and MQ contributions. The dashed vertical line marks the selected wavelength λ_sel_^PW,0^ = 425 nm. (b) Spectral evolution of the quantities entering the lossless selection metric: MD purity, absolute ED suppression, EQ suppression, MQ suppression, and the normalized total score. For the selected candidate (*n*_core_ = 4.148, *n*_shell_ = 1.774, *r* = 124.2 nm, and *R* = 231.7 nm), the selected state occurs at 425.0 nm with *Q*_ED_ = 0.0010, *Q*_MD_ = 0.2214, *Q*_EQ_ = 0.0015, and *Q*_MQ_ = 0.0094. (c) ED and MD scattering efficiencies of the selected core–shell nanoparticle for the lossless case (solid lines) and for a weakly lossy counterpart (dashed lines). The lossless state corresponds to the candidate selected in figure, with *n*_core_ = 4.147, *n*_shell_ = 1.773, *r* = 124.199 nm, and *R* = 231.72 nm. The weakly lossy case keeps the same geometry and real refractive indices, while using the best nonzero-loss counterpart from the same-geometry loss scan, with *k*_core_ = 0.0 and *k*_shell_ = 0.007143. The vertical dashed line marks the selected plane-wave wavelength, λ_sel_^PW,0^ = 425 nm. The spectra show that the selected state is MD-favored and remains identifiable after the introduction of finite loss. (d) Cartesian ED and TD contributions, plotted as normalized |*P*| and |i*kT*|, for the same structure in the lossless and weakly lossy cases. Their near-equality around the selected wavelength confirms the anapole-assisted cancellation underlying the reduced ED response. (e) Comparison of the normalized ED scattering efficiency, *Q*_ED_/*Q*_ED_^max^, and the mismatch parameter Δ_PT_ = |*P* + i*kT*|/(|*P*| + |i*kT*|) for the lossless and weakly lossy cases. The close correspondence between the minima of *Q*_ED_ and Δ_PT_ confirms that the suppressed ED channel is associated with *P*–*T* cancellation rather than with simple magnetic-mode overshadowing. For the lossless case, the mismatch minimum occurs at 425.0 nm, while the absolute *Q*_ED_ minimum occurs at 426.5 nm; the weakly lossy case shows the same trend with slightly increased residual mismatch.

The central question, however, is whether the reduced ED contribution arises from genuine anapole-assisted cancellation or simply from the relative growth of the MD channel.^24^ Figure 2c compares the ED and MD spectra of the selected particle for the lossless case and for a lossy counterpart defined from the same geometry and the same real refractive indices. To obtain this lossy case, we fixed the lossless candidate from Figure 2a,b and then scanned the (*k*_core_ and *k*_shell_) plane for that same geometry, selecting the best nonzero-loss point using the same screening criterion (see Figure S2). This yielded *k*_core_ = 0 and *k*_shell_ = 0.007143. The lossy case therefore functions as a robustness test of the intrinsic lossless benchmark. The spectra show that the MD-favored state remains centered near the selected wavelength; dissipation mainly reduces the contrast and slightly broadens the response without changing the low-order spectral ordering. The decisive evidence is provided by the Cartesian-dipole analysis in Figure 2d,e. Figure 2d shows the normalized amplitudes |*P*| and |i*kT*| for the lossless and lossy cases. Around the selected wavelength, the electric and toroidal contributions become nearly equal, indicating the cancellation condition associated with an anapole-like ED suppression. This interpretation is reinforced by Figure 2e, where the normalized spherical ED response *Q*_ED_/*Q*_ED_^max^ is compared with the mismatch parameter, $\Delta_{PT}=\frac{\left|P+ikT\right|}{\left|P\right|+\left|ikT\right|}$ . For the lossless case, the mismatch minimum occurs at 425.0 nm, while the absolute *Q*_ED_ minimum occurs at 426.5 nm. The lossy case shows the same ordering, with a slightly larger residual mismatch. The close correspondence between the minima of *Q*_ED_ and Δ_PT_ demonstrates that the reduced ED channel is linked to *P*–*T* cancellation rather than to simple magnetic overshadowing. In other words, the selected plane-wave state is MD-favored in the spherical multipole picture and carries the Cartesian signature expected for anapole-assisted ED suppression.

The full low-order multipolar comparison between the lossless and lossy cases is provided in Figure S3, confirming that the weak-loss perturbation preserves the same ED-suppressed, MD-favored low-order hierarchy observed in the main-text spectra. At the same time, the plane-wave route also reveals its own limitation. Although the selected state is MD-favored in the low-order decomposition and is accompanied by anapole-assisted ED reduction, this does not automatically imply that the full far-field radiator is equivalent to an ideal pure MD source. Figure S4a shows the normalized angular pattern of a pure MD reference, while Figure S4b shows the far-field pattern of the selected core–shell particle at λ_sel_^PW,0^ = 425.0 nm for both the lossless and lossy cases. The pattern comparison in Figure S4c makes clear that the selected particle does not reproduce the pure-MD angular distribution exactly. The deviation remains substantial, with RMS = 0.630 and correlation = 0.622 for the lossless case, and RMS = 0.637 and correlation = 0.604 for the lossy case. Thus, even though the low-order spectral response is strongly MD-favored, the full radiation pattern still differs appreciably from that of an ideal MD radiator. The origin of this discrepancy is clarified by the modal decomposition in Figure S4d. At the selected wavelength, the ED, MD, EQ, and MQ channels are all individually small compared with the total HO background. This means that the low-order MD dominance identified in Figure 2 does not translate into full far-field purity because the residual HO content remains significant. The plane-wave route therefore provides an important while incomplete benchmark: it can identify a state with strong MD character and can verify that the suppressed ED response is tied to anapole-assisted *P*–*T* cancellation, yet the resulting radiator is still not clean in the full far-field sense.

Thus, the plane-wave branch establishes the intrinsic optical benchmark of the selected core–shell design and confirms that the reduced ED channel has an anapole-related origin. At the same time, this shows that plane-wave excitation does not by itself produce a clean far-field MD radiator because HO radiation remains significant. This limitation motivates the aloof-electron CL analysis, where the question becomes whether a localized electron beam can condition the radiative multipolar output more selectively.

### Aloof-Electron CL Access to Magnetic-Dipole-Favored Emission

Having established the plane-wave benchmark and its far-field limitation, we next ask whether aloof-electron excitation can access a more selective magnetic-dipole-favored radiative response. A plane wave probes the particle through a spatially extended transverse field, whereas a fast electron excites it through a localized evanescent field whose spectral and angular-momentum content depends on the electron velocity (β) and impact parameter (*b*).^16,17,25^ The optimum under aloof excitation therefore need not coincide with the plane-wave-selected structure. We treat the aloof-electron branch as a separate source-conditioned selection problem within the same core–shell design space. The search procedure is summarized in Figure 3. We use the MD cathodoluminescence purity, $\eta_{MD}^{CL}=\frac{\Gamma_{MD}^{CL}}{\Gamma_{CL}}$ together with a composite selection score that rewards a finite MD cathodoluminescence contribution while penalizing ED and HO leakage. The full expression for the score and the numerical workflow is given in Section S2 of the Supporting Information. This distinction is important because purity alone can become misleading when the total radiative signal is very small.

**Figure 3:**
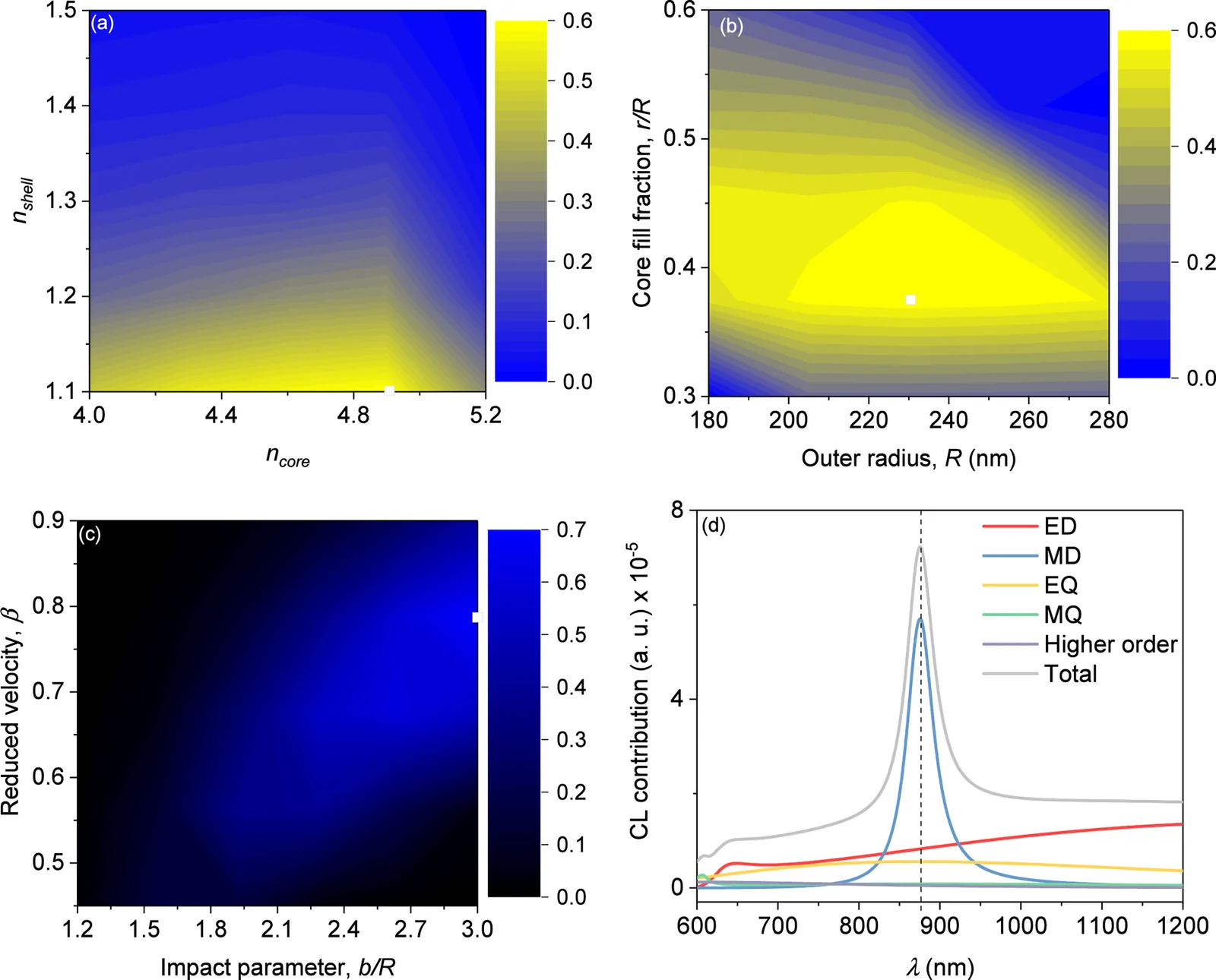
(a) Index-space map showing the best aloof-electron CL selection score obtained at each (*n*_core_ and *n*_shell_) pair by optimizing the wavelength for a fixed geometry (*R* = 260 nm and *r*/*R* = 0.45) under the representative aloof condition (β = 0.695 and *b*/*R* = 2.5). The white square marks the selected synthetic core–shell index pair, *n*_core_ = 4.90 and *n*_shell_ = 1.05. (b) Geometry-space map of the best selection score for the index pair chosen in panel (a) obtained by optimizing the wavelength over the (*R*, *r*/*R*) space under the same representative beam condition. The white square marks the selected geometry, *R* = 230 nm and *r*/*R* = 0.375, corresponding to *r* = 86.2 nm. (c) Beam-space map of the best selection score for the candidate selected in panels (a,b) showing the dependence of the CL response on reduced electron velocity β and normalized impact parameter *b*/*R*. The white square identifies the final operating point, β = 0.788 and *b*/*R* = 3.00, at which the MD-favored CL state is obtained at λ_MD_^CL^ = 875 nm. In all panels, the color bars represent the best selection score. (d) Multipolar aloof-electron CL spectrum at the selected operating point, decomposed into ED, MD, EQ + MQ, HO, and total contributions. The spectrum shows that the selected condition is dominated by the MD, with a MD purity of η_MD_^CL^ = Γ_MD_^CL^/Γ_CL_ ≈ 0.795. The selection score used in panels (a–c) rewards a visible MD contribution while penalizing ED and HO leakage.

Figure 3a maps the best aloof-CL selection score in synthetic index space. In this first step, the geometry is fixed at *R* = 260 nm and *r*/*R* = 0.45, and the representative aloof condition is set to β = 0.695 and *b*/*R* = 2.5. The scan is restricted to *n*_shell_ > 1, so that the shell remains formally distinct from the air background. The selected index pair is *n*_core_ = 4.90 and *n*_shell_ = 1.05, with the strongest score occurring near λ = 1175 nm. Because this shell index lies close to the lower bound of the synthetic search, it should be interpreted as a limiting weak-shell design point rather than as a direct material prescription. The high-index core selected in this synthetic map should be read in the same way: it identifies the strongest limiting case within the scanned parameter space, not a strict material requirement. The real-material Si@MgF_2_ calculation in Figure 5 tests whether the same design rule survives in a practical silicon-based dielectric system. Within the present synthetic search window, the optimum therefore indicates a preference for a high-index core and a weakly perturbing low-index shell; the real-material translation in Figure 5 tests this tendency using a physically plausible solid shell. Using this selected index pair, Figure 3b searches the geometry space. The optimum shifts to a smaller outer radius and core filling fraction, giving *R* = 230 nm and *r*/*R* = 0.375, corresponding to *r* = 86.2 nm. At the representative beam condition used for structural selection, this geometry gives η_MD_^CL^ ≈ 0.697 near λ = 875 nm. This step shows that the index-selected structure is not automatically the geometry-selected structure; the aloof response is set jointly by refractive-index contrast, modal size, and electron-field overlap.

The final beam-conditioned step is shown in Figure 3c. Here the selected core–shell particle from (Figure 3a,b) is fixed, while the reduced electron velocity and normalized impact parameter are varied. The best operating point in the scanned beam space occurs at β = 0.788 and *b*/*R* = 3.00, again at λ_IMD_^CL^ = 875 nm. This result shows that the MD-favored CL response is not determined by the particle alone. It is also conditioned by the electron trajectory and velocity, which control how the passing charge samples the multipolar response.^18,20^ Figure 3d shows the corresponding multipolar CL spectrum. At the selected operating point, the MD contribution is the dominant radiative channel, while the ED, quadrupolar, and HO contributions remain comparatively weak. The resulting MD purity is η_MD_^CL^ ≈ 0.795. Figure 3 therefore establishes a single internally consistent synthetic aloof-electron benchmark: *n*_core_ = 4.90, *n*_shell_ = 1.05, *R* = 230 nm, *r*/*R* = 0.375, β = 0.788, *b*/*R* = 3.00, and λ_IMD_^CL^ = 875 nm.

We next test whether this selected point is a robust operating condition rather than an artifact of the finite search grid. Figure 4a extends the (λ,*b*/*R*) map for the same locked candidate at fixed β = 0.788. Figure 3 operating point lies close to the maximum of the extended map. The highest score in the enlarged search occurs at *b*/*R* ≈ 3.33, with nearly the same MD purity, η_MD_^CL^ ≈ 0.798, as Figure 3 point. Thus, the selected operating point is not a spurious boundary artifact of the initial beam-space scan; it lies within a finite high-score region of the aloof-electron operating window. Figure 4b clarifies the physical meaning of this operating window through a line cut at λ = 875 nm. As *b*/*R* increases, the electron moves farther from the particle, and the near-field coupling strength decreases, reducing both the total CL yield and the absolute MD CL yield. At the same time, the modal selectivity remains high over the same region. The selected point at *b*/*R* = 3.00 therefore represents a yield-selectivity compromise rather than a purely purity-driven limit. This is the key operational distinction of the aloof-electron route: increasing the aloof distance can preserve MD selectivity, however it also reduces the measurable radiative signal.

**Figure 4:**
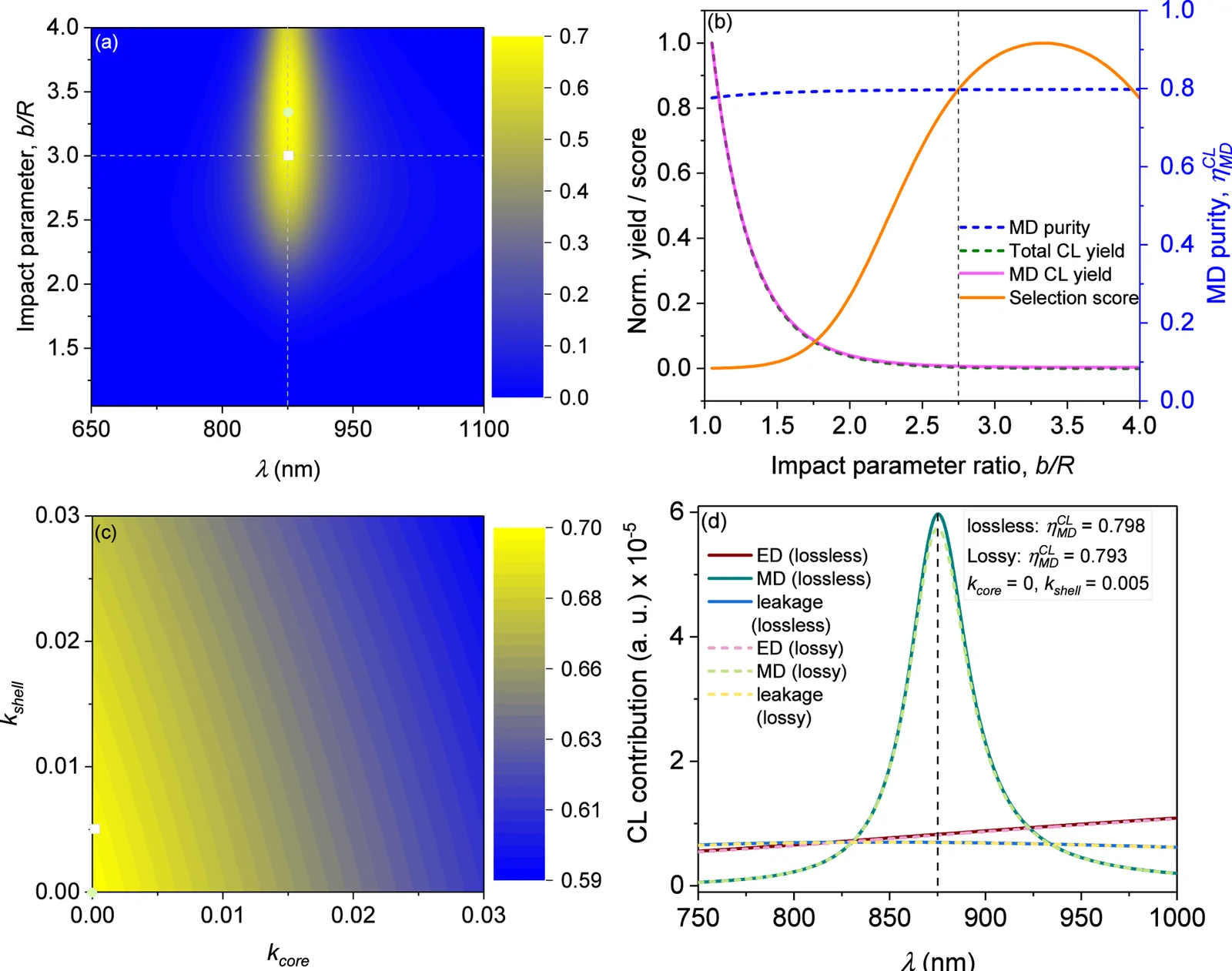
(a) Extended wavelength-impact-parameter (λ,*b*/*R*) map of the aloof-electron CL selection score for the synthetic core–shell candidate identified in Figure 3, with *n*_core_ = 4.90, *n*_shell_ = 1.05, *R* = 230 nm, and *r*/*R* = 0.375, at fixed reduced electron velocity β = 0.788. The white square marks the operating point selected in Figure 3, λ_IMD_^CL^ = 875 nm and *b*/*R* = 3.00, while the open circle marks the maximum found in the extended map. (b) Line cut at λ = 875 nm showing the dependence of the normalized total CL yield, normalized MD CL yield, normalized selection score, and MD purity η_MD_^CL^ = Γ_MD_^CL^/Γ_CL_ on the aloof distance *b*/*R*. The selected operating point provides a physically meaningful compromise between MD selectivity and measurable radiative signal, illustrating the expected trade-off between increasing modal selectivity and decreasing overall excitation strength as the beam moves farther from the particle. (c) Loss scan in the (*k*_core_ and *k*_shell_) plane for the same structure and beam condition, with the wavelength reoptimized at each loss pair. The circle denotes the lossless reference point, and the white square marks the best nonzero-loss point. (d) Multipolar aloof-electron CL spectra comparing the lossless candidate with the selected lossy counterpart. The dominant MD contribution remains intact, whereas the ED and HO leakage channels remain comparatively weak. The persistence of the spectral hierarchy confirms that the selected aloof-electron MD-favored state is robust against weak material loss.

Finally, Figure 4c,d examines loss robustness for the same structure and beam condition. In Figure 4c, the geometry, real refractive indices, β, and *b*/*R* are fixed, while the extinction coefficients (*k*_core_ and *k*_shell_) are scanned and the wavelength is reoptimized at each loss pair. The lossless point gives η_MD_^CL^ ≈ 0.798. The best nonzero-loss point occurs at *k*_core_ = 0 and *k*_shell_ = 0.005, with η_MD_^CL^ ≈ 0.793. The small reduction in purity shows that the selected MD-favored CL state is not produced by absorption and is not immediately destroyed by weak dissipation. The spectra in Figure 4d provide the corresponding multipolar confirmation. The lossy counterpart retains the same qualitative hierarchy as the lossless structure: the MD channel remains dominant, while ED and HO leakage remain smaller. Thus, the aloof-electron result is not a single-point numerical optimum. It persists over a finite operating window, follows the expected yield-selectivity trade-off with aloof distance, and remains stable under a small dissipative perturbation. Together, Figures 3 and 4 identify aloof-electron CL as a source-conditioned route for accessing MD-favored radiative emission in a core–shell nanoparticle.

### Real-Material Translation of the Aloof-Electron Response

The preceding analysis used synthetic, lossless refractive indices to isolate the source-conditioned selection physics. As a final step, we ask whether the same aloof-electron design tendency can survive when a realistic solid-shell material pair is introduced. This step also addresses the practical index limitation of the synthetic search. Although the strongest synthetic score occurs for a very high-index core, silicon provides a realistic dielectric platform in the near-infrared range considered here. This step is not intended to provide an exhaustive materials survey. Instead, it tests whether the magnetic-dipole-favored response identified in the synthetic parameter space can be recovered, in reduced form, using a physically plausible dielectric core–shell system. Figure 5a shows the multipolar aloof-electron CL spectrum of a Si@MgF_2_ core–shell particle with *r* = 100 nm and *R* = 120 nm, using tabulated dispersive optical constants for Si and MgF_2_,^26,27^ and excited at *b*/*R* = 2.5. The selected wavelength is λ = 895 nm. At this point, the modal fractions are approximately ED = 0.260, MD = 0.729, and leakage = 0.011, where leakage denotes the combined EQ, MQ, and HO contributions. Thus, unlike the earlier less favorable real-material candidates, the compact Si@MgF_2_ geometry gives a clearly magnetic-dipole-favored CL response: the MD contribution is substantially larger than the ED contribution, while HO contamination remains weak.

**Figure 5:**
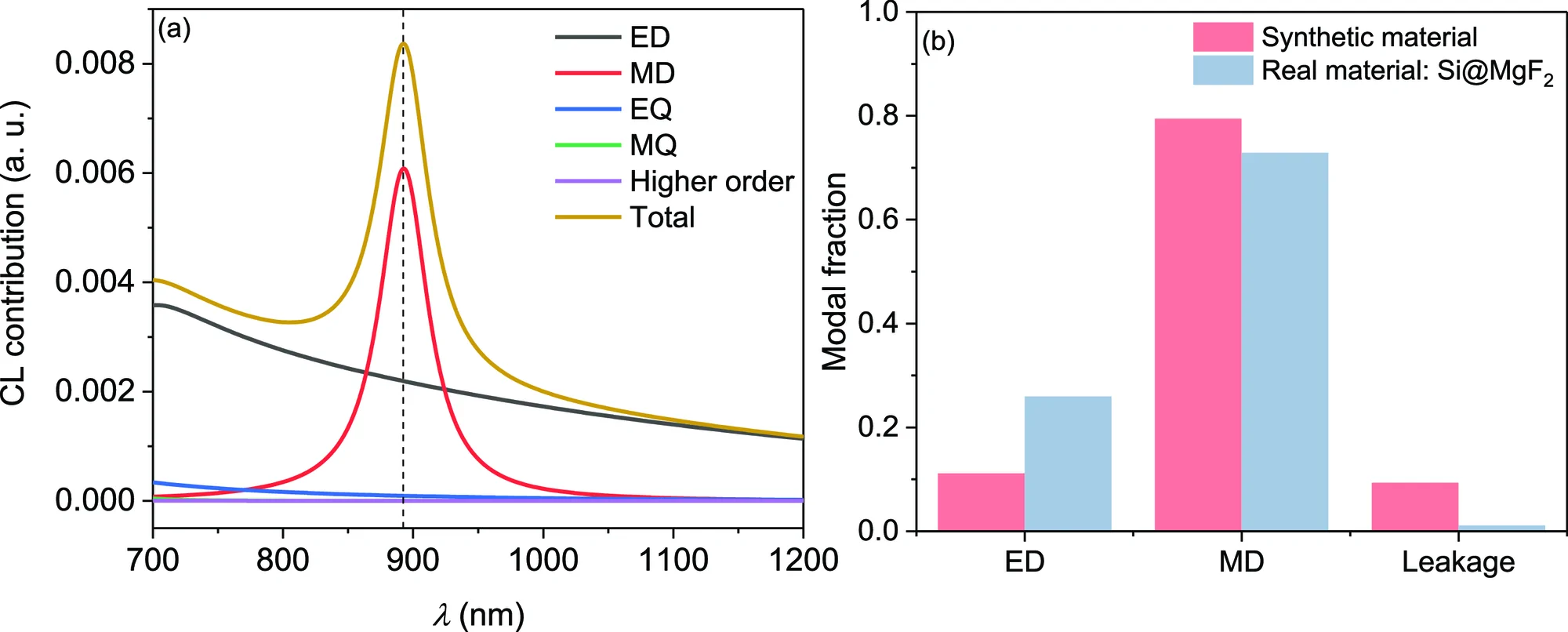
(a) Multipolar aloof-electron CL spectrum of the selected real-material core–shell candidate, Si@MgF_2_, with *r* = 100 nm, *R* = 120 nm, and *b*/*R* = 2.5. The dashed line marks the selected operating wavelength, λ = 895 nm. At this wavelength, the modal fractions are ED = 0.260, MD = 0.729, and leakage = 0.011, where leakage denotes the combined EQ, MQ, and HO contribution. (b) Modal-fraction comparison between the locked synthetic aloof benchmark from Figure 3 and the selected real-material Si@MgF_2_ candidate.

The comparison in Figure 5b places this real material response next to the synthetic aloof benchmark from Figure 3. As expected, the real material system does not reproduce the ideal synthetic selectivity exactly. The ED fraction increases from approximately 0.112 in the synthetic benchmark to approximately 0.260 in the Si@MgF_2_ candidate, while the MD fraction decreases from approximately 0.795 to approximately 0.729. This increase in ED contribution is expected because replacing the near-air synthetic shell with a finite-index dispersive MgF_2_ shell modifies the core–shell polarizability and the beam-weighted modal overlap, partially lifting the near-ideal ED suppression while leaving the MD channel dominant. However, the hierarchy remains favorable: the MD channel is still the largest radiative contribution and the HO leakage remains small. This indicates that the aloof electron selection rule identified in the synthetic study is not limited to an artificial parameter set, although its performance is reduced by realistic dispersion and material contrast. This real-material example should therefore be viewed as a proof-of-principle translation rather than a final material optimization. The key result is that a realistic low-loss dielectric core–shell pair can preserve the qualitative synthetic design rule: a properly conditioned aloof electron can favor magnetic-dipole CL while suppressing HO leakage. Together with Figures 3 and 4, this supports the central conclusion that MD-favored emission is governed by both particle geometry and the excitation source and trajectory.

## Discussion

The results show that the MD-favored response in a core–shell nanoparticle is not determined by geometry alone. It is jointly controlled by material contrast, modal interference, and the spatial structure of the excitation source. Under plane-wave illumination, the selective MD response requires simultaneous enhancement of the MD channel and suppression of the competing ED and HO channels at the same operating wavelength. The plane-wave analysis therefore provides a necessary benchmark: it identifies an MD-favored scattering condition and confirms that the reduced ED contribution is linked to anapole-assisted cancellation between the Cartesian electric and toroidal dipoles, rather than to a trivial increase of the MD peak alone. This distinction is important because a large MD resonance by itself does not guarantee MD selectivity.

The relation to simple aspect ratio or shape-tuning design rules should therefore be clarified. Such rules are valuable for shifting electric and magnetic Mie resonances and for engineering directional scattering under optical excitation. The present problem is different because the selected observable is a beam-weighted CL hierarchy rather than a structural resonance wavelength alone. The optimum depends simultaneously on the core–shell Mie spectrum, ED suppression, HO leakage, electron velocity, and impact parameter. An aspect ratio rule can guide the placement of magnetic resonances, whereas MD-dominant radiative CL under aloof-electron excitation requires the proper beam-weighted balance among ED, MD, and leakage channels. Moreover, the present result is also connected to recent work on ideal magnetic light emission from Mie nanoparticles coupled to localized optical emitters.^28^ In both cases, a localized source weights the Mie channels differently from plane-wave excitation and can enhance the MD contribution to the emitted field. The distinction is that an optical emitter is a fixed dipolar source controlled by position, orientation, and transition frequency, whereas an aloof electron is a moving broadband evanescent source controlled by the velocity and impact parameter.

The plane-wave search was deliberately initiated in the lossless limit so that ED suppression could be attributed to modal interference rather than to absorption-induced damping; finite loss was introduced only afterward as a perturbative robustness test. The aloof-electron results add a second level of control. Unlike a plane wave, a fast electron excites the particle through a localized evanescent field whose spectral and angular-momentum content depends on the electron velocity and impact parameter. The electron trajectory therefore becomes part of the modal selection mechanism. Figures 3 and 4 show that the selected CL response is not fixed only by the core–shell geometry: the same particle can give different multipolar hierarchies as β and *b*/*R* are varied. This source-conditioned behavior is one of the central findings of the work. This suggests that aloof-electron excitation can serve as a probe of optical resonances and as a selective driver of radiative multipolar channels.

We do not use a separate Cartesian *P*–*T* reconstruction as the defining diagnostic for the aloof-optimized state because the aloof section addresses a beam-weighted radiative readout rather than a new current-level anapole condition. The relevant observable is therefore the CL multipolar hierarchy, Γ_MD_^CL^, Γ_ED_^CL^, and HO leakage, while the plane-wave *P*–*T* analysis serves as the intrinsic optical benchmark. A key point is that the aloof-electron optimum is not merely a single numerical point. The extended (λ,*b*/*R*) map in Figure 4 shows that the selected state lies within a finite operating window, rather than in an isolated accidental condition. The nearby maximum in the enlarged search confirms that the Figure 3 operating point is not a boundary artifact of the initial scan. At the same time, the line cut in Figure 4b shows the physical cost of increasing the aloof distance: modal selectivity can be preserved, whereas the total CL yield and absolute MD yield decrease as the electron moves farther from the particle. The selected condition should therefore be understood as a yield-selectivity compromise, not as a purely purity-maximized limit. This is an important practical message for experiments, where the observable CL signal is governed by modal purity, beam current, collection efficiency, detector response, and sample stability.^15–20^

The angular balance of the selected aloof electron CL states also affects experimental collection. We therefore added a forward/backward hemispheric analysis in the Supporting Information (S5.1). Here, *F* and *B* denote the integrated CL intensities emitted into the forward and backward hemispheres, respectively, defined relative to the electron propagation axis (+*z*). The synthetic aloof-electron benchmark shows a modest forward bias with *F* = 0.579, *B* = 0.421, and *A*_FB_ = 0.157. The real-material Si@MgF_2_ candidate shows a stronger forward bias with *F* = 0.692, *B* = 0.308, and *A*_FB_ = 0.383. Thus, the selected CL states are preferentially forward-emitting, especially for the Si@MgF_2_ candidate; however, they are not purely unidirectional. In a substrate-supported experiment, the forward component may partly couple into the substrate and may be reduced in an air-side collection geometry, whereas the finite backward component remains directly accessible. Depending on the substrate reflectivity and collection geometry, part of the forward-directed radiation may also be indirectly recovered through reflections. This analysis provides the intrinsic free-space angular balance of the selected CL states; a full substrate-resolved, angle-resolved collection model is outside the scope of this work.

Penetrating trajectories lie outside the present optimization because they change the excitation problem rather than simply extend the aloof-distance scan. In the aloof regime, the electron drives the particle through its external evanescent field, allowing the radiative multipolar hierarchy to be analyzed without a direct passage through the structure. Once the beam enters the particle, interface crossing and internal excitation can introduce transition radiation, Cherenkov-type, and bulk loss contributions; near-central trajectories can also suppress magnetic circulating current excitation by symmetry. Direct passage further increases the relevance of dose deposition, charging, heating, contamination, and beam-induced structural modifications. A full aloof-to-penetrating comparison therefore requires a separate treatment, as also supported by recent analytical developments for core–shell nanoparticles under fast-electron excitation.^29^ The present work focuses on aloof/off-axis excitation as the cleaner configuration for identifying MD-favored radiative CL emission, while penetrating trajectories are left as an important direction for future studies.

The small-loss analysis shown in Figure 4 should be interpreted as a controlled perturbation test. The nonzero-loss point with *k*_shell_ = 0.005 is not intended to represent a specific fitted shell material. Instead, it asks whether the selected aloof-electron MD-favored state collapses immediately when weak dissipation is introduced. The answer is the modal hierarchy, and MD purity changes only slightly. This behavior is physically reasonable since increasing absorption generally damps the resonant response and reduces the selection score. The role of Figure 4 is therefore to establish robustness against a small dissipative perturbation, while Figure 5 separately addresses a realistic material translation with dispersive optical constants.

The real-material calculation confirms both the promise and constraint of the synthetic design. In the idealized synthetic case, the MD fraction is high, and the ED channel is strongly suppressed. When a realistic low-loss dielectric pair is introduced, the response is reduced; however, it is not entirely lost. The compact Si@MgF_2_ candidate in Figure 5 retains an MD-favored CL response, with the MD contribution remaining clearly larger than the ED contribution and with very small HO leakage. This result is not presented as a global material optimum. It shows, however, that a core index above 4 is not a strict requirement for obtaining a magnetic dipole-favored CL hierarchy; realistic silicon-based dielectric structures can retain the effect, although with reduced selectivity compared with the synthetic optimum. Rather, it demonstrates that the source-conditioned selection rule identified in synthetic space can survive, in reduced form, in a physically plausible core–shell system. Further optimization over material dispersion, shell thickness, beam energy, and impact parameter may improve the real-material performance.

Several limitations must be kept in view. First, synthetic screening is finite and is designed to expose the electromagnetic mechanism, not to prove global optimality over all possible refractive indices, radii, and beam conditions. Second, the real-material example is a first translation based on representative dispersive optical constants; more complete material data sets and fabrication-specific optical constants could shift the selected wavelength and modal fractions. Third, the calculations consider an isolated spherical core–shell particle and an ideal aloof trajectory. Substrate effects, finite collection angles, surface roughness, size dispersion, beam-induced damage, and detector response are not included in the present model. These factors will affect the absolute CL count rates and experimental visibility. They do not, however, change the central conclusion, which concerns the relative multipolar hierarchy and its dependence on the excitation source. Overall, these results identify MD-favored response as a source-conditioned property of the core–shell nanosystem rather than a geometry-only feature. The plane-wave analysis establishes the intrinsic optical benchmark through anapole-assisted ED suppression, while the aloof-electron analysis shows that the electron velocity and impact parameter can reshape the radiative multipolar hierarchy. The real material Si@MgF_2_ example indicates that this design tendency can persist beyond the idealized synthesis parameter space.

Electron beam-induced damage is another practical limitation. A quantitative damage assessment would require a dose-dependent material model including beam current, dwell time, acceleration voltage, heat dissipation, charging, contamination, and possible structural or chemical modification, which is outside of the present electromagnetic treatment. The aloof geometry is favorable in this respect because it avoids direct electron passage through the nanoparticle and reduces the direct energy deposition relative to penetrating trajectories. Practical implementation would therefore require low-dose CL conditions and the optimization of beam current, dwell time, acceleration voltage, collection efficiency, and sample stability.

## Conclusions

We show that the MD-favored response in core–shell nanoparticles is governed by the combined action of core–shell geometry, material contrast, and the aloof-electron excitation source. Under plane-wave illumination, the key requirement is simultaneous MD enhancement and ED suppression, with the reduced ED channel traced to anapole-assisted electric-toroidal dipole cancellation. Under aloof-electron excitation, the electron velocity and impact parameter provide additional control over the radiative multipolar hierarchy, enabling a MD-favored CL state that persists over a finite operating window and remains robust against weak loss. A real-material Si@MgF_2_ example shows that the same design tendency survives, in reduced form, under realistic dispersion and material contrast. These results provide a source-conditioned design principle for selecting MD-favored scattering and CL in nanoscale core–shell Mie resonators.

## Methods

This work is a theoretical and computational study of concentric core–shell nanospheres under plane-wave and aloof-electron excitation. The calculations were carried out with in-house Python routines using a semianalytical multipolar formulation. The implementation uses analytic core–shell Mie coefficients for the optical response and beam-weighted spherical multipole channels for the aloof electron cathodoluminescence response. Detailed expressions for multipolar decomposition, purity definitions, and anapole diagnostics are provided in Section S1 of the Supporting Information. The parameter searches were organized into separate plane-wave and aloof-electron branches. The plane-wave branch was used to establish an intrinsic optical benchmark, while the aloof-electron branch was used to identify beam-conditioned radiative cathodoluminescence states. Candidate structures were selected using composite metrics that favor the MD response and penalize residual ED, quadrupolar, and HO leakage channels. The full selection metrics, scan workflow, and numerical ranges are given in Section S2 of the Supporting Information. The term numerical refers to wavelength scans, parameter-space searches, and finite multipole summations within the semianalytical model. The relevant numerical controls are the multipole cutoff, wavelength resolution, and parameter-grid resolution. Additional implementation details and convergence controls are provided in Section S1.1 of the Supporting Information. The loss-robustness tests, angular-balance analysis, and real-material Si@MgF_2_ translation are described in Sections S3–S6 of the Supporting Information.

## Supplementary Material



## Data Availability

The data supporting the plots within this paper and other study findings are available from the corresponding author upon reasonable request.

## Notes

Semianalytical computational implementation and convergence controls; multipolar decomposition; selection metrics and numerical workflow; plane-wave synthetic search and weak-loss perturbation; far-field angular-pattern comparison; aloof-electron numerical implementation; aloof-electron angular-balance analysis; and real-material Si@MgF_2_ candidate parameters and modal fractions (PDF)
