# Supplementary material for: Electron-Beam-Conditioned Magnetic-Dipole Emission in Cathodoluminescence
Source: ACS Nanosci Au. 2026 Jul 7;6(5):903–12. doi: 10.1021/acsnanoscienceau.6c00074 (PMC13693251; doi:10.1021/acsnanoscienceau.6c00074)
Supplement: Supplementary file 1 [file ng6c00074_si_001.pdf]

Supporting Information for "Electron-Beam-Conditioned Magnetic-Dipole Emission in Cathodoluminescence"

Gour Mohan Das[1†*], Kameyab Raza Abidi[2†], and Govindan Vadivel[3]

[1]*Nanoscience Center, Department of Chemistry, University of Jyväskylä, Jyväskylä, 40014 Finland*

[2]*Nanoscience Center, Department of Physics, University of Jyväskylä, Jyväskylä, 40014 Finland*

[3]*Functional Material Technology Group, Ensemble[3] Centre of Excellence, Wólczyńska 133, Warsaw, 01-919 Poland*

†Equal Contribution

*gour.m.das@jyu.fi

S1. Computational framework and multipolar decomposition

We consider a concentric core-shell nanosphere embedded in air. The core radius is r, the outer radius is R, and the shell thickness is $t = R - r$. The core and shell are described by complex refractive indices

$$m_{\mathrm{core}}(\lambda) = n_{\mathrm{core}}(\lambda) + ik_{\mathrm{core}}(\lambda),$$
$$m_{\mathrm{shell}}(\lambda) = n_{\mathrm{shell}}(\lambda) + ik_{\mathrm{shell}}(\lambda),$$

respectively. The surrounding medium is air, $m_{\mathrm{h}} = 1$, and all media are treated as nonmagnetic. The free-space wavenumber is $k_0 = 2\pi/\lambda$, where λ is the vacuum wavelength. The optical response of the core-shell particle was evaluated using standard core-shell Mie theory for concentric spheres [1]. The electric and magnetic spherical Mie coefficients are denoted by a_ℓ and b_ℓ, respectively. For plane-wave excitation, the total scattering efficiency was decomposed into ED, MD, EQ, MQ, and HO contributions,

$$Q_{\mathrm{tot}} = Q_{\mathrm{ED}} + Q_{\mathrm{MD}} + Q_{\mathrm{EQ}} + Q_{\mathrm{MQ}} + Q_{\mathrm{HO}}.$$

Here $Q_{\rm ED}$ and $Q_{\rm MD}$ correspond to the $\ell = 1$ electric and magnetic channels, $Q_{\rm EQ}$ and $Q_{\rm MQ}$ correspond to the $\ell = 2$ electric and magnetic channels, and $Q_{\rm HO}$ collects all HO contributions with $\ell \geq 3$. The plane-wave MD purity is defined as

$$\eta_{\rm MD}^{\rm PW} = \frac{Q_{\rm MD}}{Q_{\rm tot}}.$$

A large value of $\eta_{\rm MD}^{\rm PW}$ indicates that the MD dominates the scattering efficiency at a given wavelength. However, this quantity alone does not prove an ideal MD response, because the relative MD fraction can increase simply when the total scattering is dominated by a strong MD resonance. Therefore, the plane-wave analysis also monitors the absolute suppression of the electric-dipole and quadrupolar channels.

To verify whether the suppressed ED channel is related to anapole-assisted cancellation, the spherical multipole analysis was supplemented by a Cartesian dipole diagnostic [2,3]. The Cartesian electric dipole P and toroidal dipole T were used to evaluate the normalized mismatch parameter

$$\Delta_{PT} = \frac{| P + ik_0 T |}{| P | + | ik_0 T |}.$$

The anapole-assisted ED suppression condition corresponds to $P \approx -ik_0 T$, or equivalently to a minimum of Δ_{PT}. In the main text, this diagnostic is used only for the plane-wave benchmark, where the objective is to confirm whether the reduced ED scattering channel originates from electric–toroidal dipole cancellation rather than from simple magnetic-mode overshadowing.

For aloof-electron excitation, the particle is driven by a fast electron moving outside the sphere with impact parameter $b > R$. The reduced electron velocity is $\beta = v/c$, and the Lorentz factor is

$$\gamma = \frac{1}{\sqrt{1 - \beta^2}}.$$

The electron excites the particle through a localized evanescent field, and the radiative CL response was decomposed into the same modal groups as in the plane-wave case,

$$\Gamma_{\rm CL} = \Gamma_{\rm ED}^{\rm CL} + \Gamma_{\rm MD}^{\rm CL} + \Gamma_{\rm EQ}^{\rm CL} + \Gamma_{\rm MQ}^{\rm CL} + \Gamma_{\rm HO}^{\rm CL}.$$

Here $\Gamma_{\rm ED}^{\rm CL}$ and $\Gamma_{\rm MD}^{\rm CL}$ are the electric- and magnetic-dipole CL channels, $\Gamma_{\rm EQ}^{\rm CL}$ and $\Gamma_{\rm MQ}^{\rm CL}$ are the quadrupolar channels, and $\Gamma_{\rm HO}^{\rm CL}$ contains the remaining HO radiative contribution. MD state CL purity is defined as, $\eta_{\rm MD}^{\rm CL} = \frac{\Gamma_{\rm MD}^{\rm CL}}{\Gamma_{\rm CL}}$. This quantity measures the fraction of the radiative electron-excited signal carried by the MD channel. In contrast to the plane-wave benchmark, the aloof-electron optimum is beam-conditioned: it depends not only on the core-shell geometry and material contrast, but also on the electron velocity and impact parameter. Therefore, the aloof-electron analysis identifies a radiative CL operating point through the beam-weighted modal hierarchy, rather than through a separate Cartesian P–T reconstruction. Unless otherwise stated, the electron beam was taken to be aloof, $b > R$, and the reported CL spectra are decomposed into ED, MD, EQ, MQ, and HO contributions. The selection metrics and numerical scan ranges used to identify the plane-wave and aloof-electron candidates are given in Section S2.

S1.1. Computational implementation and convergence controls

All calculations in this work were executed using in-house Python routines grounded in a semi-analytical multipolar formulation. The plane-wave response was derived from standard concentric core-shell Mie coefficients for spherical particles. The aloof-electron cathodoluminescence response was evaluated by combining these spherical electric- and magnetic-type scattering channels with beam-dependent excitation weights for a fast electron traversing outside the particle. This methodology aligns with the standard fast-electron optical excitation framework and the spherical-particle electron-beam multipolar formalism utilized for EEL and CL calculations [4-6]. Because this semi-analytical approach is inherently mesh-free, the calculations are entirely free of spatial discretization and mesh-convergence artifacts. Consequently, computational accuracy is strictly governed by three explicit numerical controls: the maximum multipole order included in the spherical expansion, the wavelength step size in the spectra, and the resolution of the scanned parameter grids. The term "numerical" throughout this work strictly denotes wavelength scans, parameter-space searches, and finite multipole summations evaluated within this exact theoretical framework.

The ED, MD, EQ, MQ, and higher-order channels were evaluated from the same truncated multipolar expansion at each wavelength and parameter point. This ensures that the reported modal fractions are internally normalized within a single calculation. Higher-order contributions were aggregated as the sum of all retained multipoles beyond the ED, MD, EQ, and MQ channels. The selected operating points were further validated through high-resolution local wavelength scans and robustness checks across loss, impact parameter, and beam velocity, as detailed in the subsequent sections.

S2. Selection metrics and numerical workflow

The candidates in the main text were selected using composite scores rather than by MD purity alone. This choice is important because a large modal fraction can be misleading if the absolute MD contribution is weak, or if the non-MD channels are only small in a relative sense. The selection scores were therefore constructed to reward three simultaneous conditions: a visible MD response, suppression of the ED channel, and suppression of quadrupolar and HO leakage.

For the plane-wave branch, the MD purity is

$$\eta_{\mathrm{MD}}^{\mathrm{PW}} = \frac{Q_{\mathrm{MD}}}{Q_{\mathrm{tot}}}.$$

The practical plane-wave selection score was defined as

$$S_{\mathrm{PW}} = \eta_{\mathrm{MD}}^{\mathrm{PW}}\, F_{\mathrm{MD}}^{\mathrm{PW}}\, F_{\mathrm{ED}}^{\mathrm{PW}}\, F_{\mathrm{EQ}}^{\mathrm{PW}}\, F_{\mathrm{MQ}}^{\mathrm{PW}},$$

where $F_{\mathrm{MD}}^{\mathrm{PW}}$ rewards a finite MD scattering contribution, and $F_{\mathrm{ED}}^{\mathrm{PW}}$, $F_{\mathrm{EQ}}^{\mathrm{PW}}$, and $F_{\mathrm{MQ}}^{\mathrm{PW}}$ penalize residual non-MD channels. In the numerical implementation, these factors were written as bounded weighting functions,

$$F_{\mathrm{MD}}^{\mathrm{PW}} = \frac{Q_{\mathrm{MD}}}{Q_{\mathrm{MD}} + Q_{\mathrm{MD}}^{(0)}},$$

$$F_{\mathrm{ED}}^{\mathrm{PW}} = \frac{1}{1 + Q_{\mathrm{ED}}/Q_{\mathrm{ED}}^{(0)}},$$

$$F_{\mathrm{EQ}}^{\mathrm{PW}} = \frac{1}{1 + Q_{\mathrm{EQ}}/Q_{\mathrm{EQ}}^{(0)}},$$

$$F_{\mathrm{MQ}}^{\mathrm{PW}} = \frac{1}{1 + Q_{\mathrm{MQ}}/Q_{\mathrm{MQ}}^{(0)}}.$$

Here $Q_{\mathrm{MD}}^{(0)}$, $Q_{\mathrm{ED}}^{(0)}$, $Q_{\mathrm{EQ}}^{(0)}$, and $Q_{\mathrm{MQ}}^{(0)}$ are reference scale factors used only to balance the numerical weights. They do not represent physical thresholds. The score is high only when the MD response is finite, the MD fraction is large, and the leading electric and quadrupolar channels remain suppressed.

For the aloof-electron CL branch, the magnetic-dipole CL purity is

$$\eta_{\mathrm{MD}}^{\mathrm{CL}} = \frac{\Gamma_{\mathrm{MD}}^{\mathrm{CL}}}{\Gamma_{\mathrm{CL}}}.$$

The corresponding CL selection score was defined as

$$S_{\mathrm{CL}} = \eta_{\mathrm{MD}}^{\mathrm{CL}}\, F_{\mathrm{MD}}^{\mathrm{CL}}\, F_{\mathrm{ED}}^{\mathrm{CL}}\, F_{\mathrm{leak}}^{\mathrm{CL}},$$

with

$$F_{\mathrm{MD}}^{\mathrm{CL}} = \frac{\Gamma_{\mathrm{MD}}^{\mathrm{CL}}}{\Gamma_{\mathrm{MD}}^{\mathrm{CL}} + \Gamma_{\mathrm{MD}}^{(0)}},$$

$$F_{\mathrm{ED}}^{\mathrm{CL}} = \frac{1}{1 + \Gamma_{\mathrm{ED}}^{\mathrm{CL}}/\Gamma_{\mathrm{ED}}^{(0)}},$$

and

$$F_{\mathrm{leak}}^{\mathrm{CL}} = \frac{1}{1 + \Gamma_{\mathrm{leak}}^{\mathrm{CL}}/\Gamma_{\mathrm{leak}}^{(0)}}.$$

The leakage channel is defined as

$$\Gamma_{\mathrm{leak}}^{\mathrm{CL}} = \Gamma_{\mathrm{EQ}}^{\mathrm{CL}} + \Gamma_{\mathrm{MQ}}^{\mathrm{CL}} + \Gamma_{\mathrm{HO}}^{\mathrm{CL}}.$$

As in the plane-wave case, the constants $\Gamma_{\mathrm{MD}}^{(0)}$, $\Gamma_{\mathrm{ED}}^{(0)}$, and $\Gamma_{\mathrm{leak}}^{(0)}$ are numerical scale factors used to prevent the score from selecting points with high purity but negligible CL signal. The purpose of S_{CL} is therefore to identify beam-conditioned operating points that combine measurable MD emission with suppressed ED and HO leakage. In all scans, the reference factors $Q^{(0)}$ and $\Gamma^{(0)}$ were kept fixed within a given scan and used only to regularize the score; the final figures report normalized scores, so the selected points are determined by relative ranking rather than by the absolute score magnitude.

The numerical workflow used in the main text is summarized in Table S1. At each step, the wavelength was optimized over the specified range and the candidate with the largest corresponding score was selected. The plane-wave search was first performed in the lossless limit in order to identify the intrinsic MD-favored benchmark without absorption-induced damping. Weak loss was introduced only after this benchmark was fixed, as a perturbative robustness test. The aloof-electron search was then carried out independently, because the electron-beam optimum depends on the beam trajectory and need not coincide with the plane-wave-selected structure.

Table S1. Numerical workflow used to select and validate the plane-wave, aloof-electron, and real-material candidates**.** The plane-wave and aloof-electron branches were treated separately because the plane wave probes the intrinsic optical benchmark, whereas the aloof electron probes a beam-conditioned radiative response. The real-material calculation was used as a compact translation test of the synthetic aloof-electron design rule.

**Stage**	**Scanned variables**	**Fixed parameters**	**Output**
Plane-wave synthetic search	λ, n_{core}, n_{shell}, r, R	$k_{\text{core}} = k_{\text{shell}} = 0$	Lossless PW candidate in Fig. 2 and Fig. S1
Plane-wave loss scan	λ, k_{core}, k_{shell}	Selected PW geometry and real refractive indices	Weakly lossy counterpart in Fig. 2(c–e), Fig. S2, and Fig. S3
Aloof index search	λ, n_{core}, n_{shell}	$R = 260\text{nm}$, $r/R = 0.45$, $\beta = 0.695$, $b/R = 2.5$	Index pair in Fig. 3(a)
Aloof geometry search	$R, \lambda, r/R$	$n_{\text{core}} = 4.90$, $n_{\text{shell}} = 1.05$, $\beta = 0.695$, $b/R = 2.5$	Geometry in Fig. 3(b)
Aloof beam search	$\beta, \lambda, b/R$	$n_{\text{core}} = 4.90$, $n_{\text{shell}} = 1.05$, $R = 230\text{nm}$, $r/R = 0.375$	Operating point in Fig. 3(c,d)
Aloof operating-window test	λ, b/R	Selected aloof candidate at fixed $\beta = 0.788$	Finite operating window in Fig. 4(a,b)
Aloof loss scan	$k_{\text{core}}, k_{\text{shell}}, \lambda$	Selected aloof candidate and beam condition	Weak-loss robustness in Fig. 4(c,d)
Real-material translation	λ	$Si@MgF_2$, $r = 100\text{nm}$, $R = 120\text{nm}$, $b/R = 2.5$	Real-material CL spectrum in Fig. 5

For the real-material translation, no global materials optimization was claimed. The $Si@MgF_2$ example was selected as a representative low-loss dielectric core-shell candidate that preserves the qualitative magnetic-dipole-favored CL hierarchy found in the synthetic aloof-electron study. The material parameters and modal fractions for this candidate are listed in Section S6.

S3. Plane-wave synthetic search and weak-loss perturbation

The plane-wave branch was used to define the intrinsic optical benchmark of the core-shell particle before considering electron-beam excitation. The search was first performed in the lossless limit, $k_{\text{core}} = k_{\text{shell}} = 0$, so that ED suppression could be attributed to modal interference rather than to absorption-induced damping. The selected lossless candidate was then tested against weak material loss by keeping the same geometry and real refractive indices fixed while scanning the extinction coefficients.

S3.1 Lossless synthetic search

Figure S1 shows the synthetic plane-wave search used to identify the lossless magnetic-dipole-favored candidate. The search varied the core and shell refractive indices together with the core radius and outer radius. At each point, the wavelength was optimized using the plane-wave selection score defined in Section S2. The selected candidate corresponds to: $n_{\text{core}} = 4.148$, $n_{\text{shell}} = 1.774$, $R = 231.7$ nm, $r = 124.2$ nm.

For this candidate, the selected plane-wave wavelength is, $\lambda_{\text{sel}}^{\text{PW},0} = 425.0$ nm. At this wavelength, the low-order multipolar scattering contributions are

$$Q_{\text{ED}} = 0.0010, Q_{\text{MD}} = 0.2214,$$
$$Q_{\text{EQ}} = 0.0015, Q_{\text{MQ}} = 0.0094.$$

Thus, the selected lossless state combines a finite MD response with strong suppression of the electric-dipole and quadrupolar channels. This lossless structure is used as the intrinsic plane-wave benchmark in the main text.

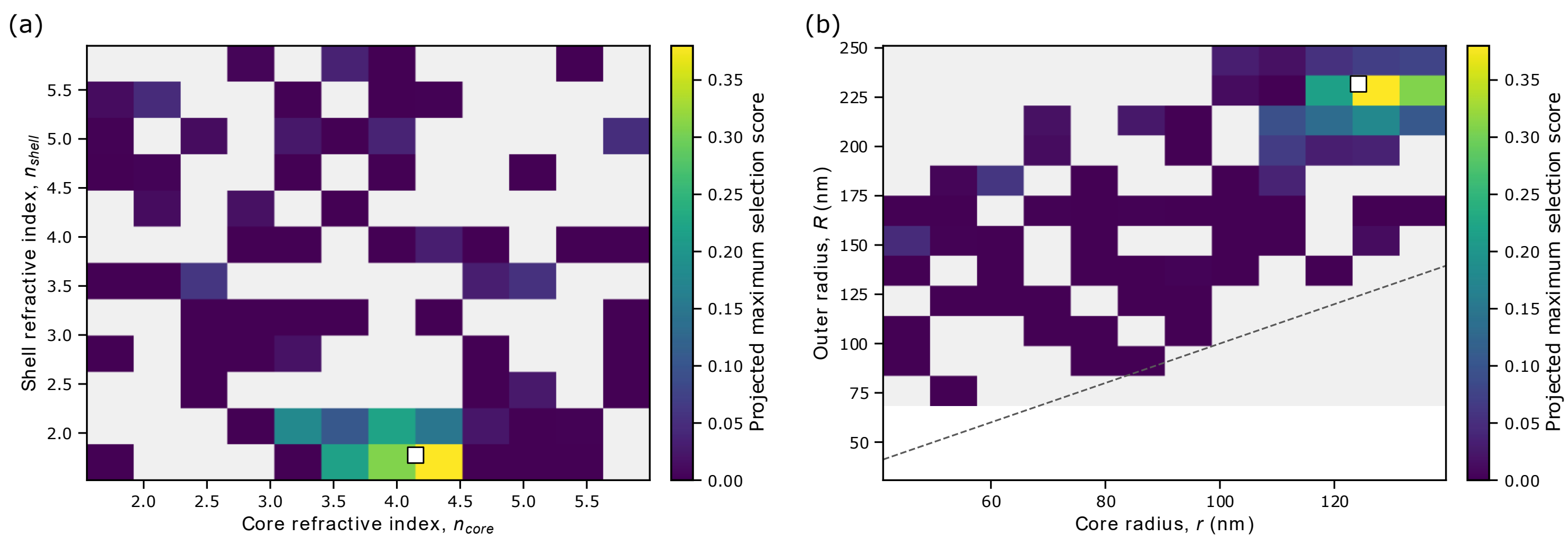

Figure S1: (a) Index-space selection map showing the best plane-wave score obtained by optimizing the wavelength and geometry for each $(n_{\text{core}}, n_{\text{shell}})$pair. (b) Geometry-space selection map for the selected index region, showing the score as a function of core radius $r$and outer radius R, or equivalently the core-shell size parameters used in the search. The white marker indicates the selected lossless candidate with $n_{\text{core}} = 4.148$, $n_{\text{shell}} = 1.774$, $r = 124.2$ nm, and $R = 231.7$ nm. The selected wavelength is $\lambda_{\text{sel}}^{\text{PW},0} = 425.0$ nm.

S3.2 Same-geometry weak-loss scan

After the lossless plane-wave candidate was fixed, a weak-loss scan was performed for the same geometry and the same real refractive indices. In this scan, only the extinction coefficients k_{core} and k_{shell} were varied, and the wavelength was re-optimized at each loss pair using the same plane-wave selection score. This procedure was used only as a perturbative robustness test; it was not intended to identify a specific material. The best nonzero-loss point from this same-geometry scan was, $k_{\text{core}} = 0, k_{\text{shell}} = 0.007143$. This weakly lossy counterpart is used in the main-text comparison of Fig. 2(c-e).

Because the geometry and real refractive indices are unchanged, any difference between the lossless and weakly lossy spectra can be attributed to the introduction of weak dissipation rather than to a change in modal structure.

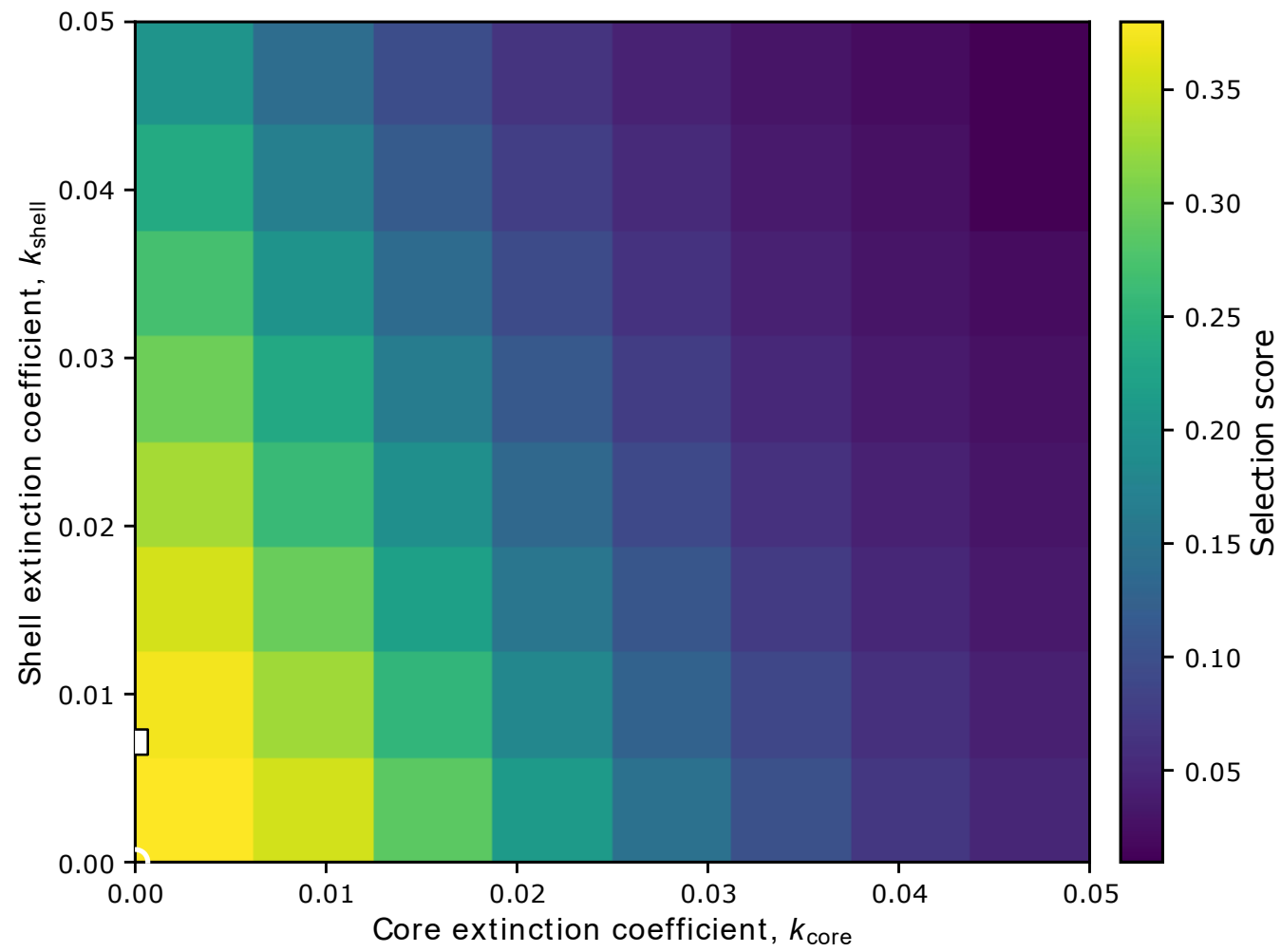

Figure S2. Plane-wave selection score plotted as a function of k_{core} and k_{shell} for the selected lossless geometry and fixed real refractive indices. The lossless point is located at $k_{\mathrm{core}} = k_{\mathrm{shell}} = 0$. The marked nonzero-loss point corresponds to $k_{\mathrm{core}} = 0$ and $k_{\mathrm{shell}} = 0.007143$, which is used as the weakly lossy counterpart in Fig. 2(c-e). This scan tests whether the MD-favored state survives a small dissipative perturbation.

S3.3 Lossless versus weakly lossy multipolar spectra

Figure S3 compares the low-order multipolar scattering spectra of the lossless benchmark and the weakly lossy counterpart. The weakly lossy case shows reduced spectral contrast and slight broadening, as expected when absorption is introduced. However, the low-order hierarchy near $\lambda_{\mathrm{sel}}^{\mathrm{PW},0} = 425.0$ nm is preserved: the ED channel remains strongly suppressed, the MD channel remains the leading low-order contribution, and the quadrupolar channels remain comparatively weak. This comparison supports the main-text conclusion that the plane-wave state is not produced by absorption. Instead, the lossless calculation identifies the intrinsic MD-favored benchmark, and the weak-loss calculation shows that this benchmark is stable against a small dissipative perturbation.

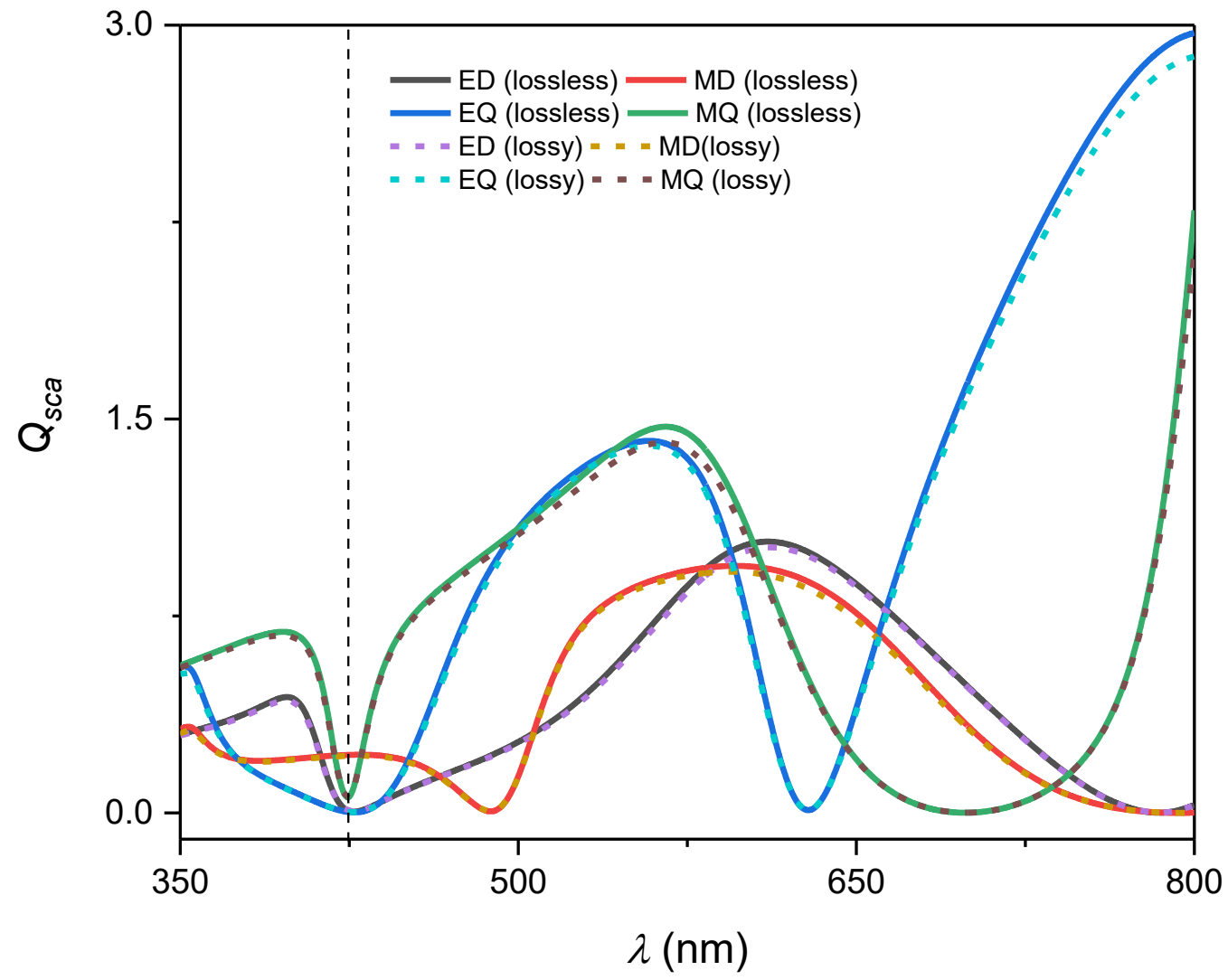

Figure S3. Comparison of the ED, MD, EQ, and MQ scattering contributions for the lossless structure and the weakly lossy counterpart. The weakly lossy case keeps the same geometry and real refractive indices as the selected lossless candidate but uses $k_{\text{core}} = 0$and $k_{\text{shell}} = 0.007143$, obtained from the same-geometry loss scan in Fig. S2. The vertical dashed line marks $\lambda_{\text{sel}}^{\text{PW},0} = 425.0$ nm.

S4. Far-field angular-pattern comparison

The plane-wave analysis in the main text identifies a low-order magnetic-dipole-favored state and confirms that the ED channel is suppressed through anapole-assisted *P*–*T* cancellation. However, low-order MD dominance in the multipolar spectrum does not automatically mean that the complete far-field radiation pattern is identical to that of an ideal pure MD. The total far field can still contain contributions from residual quadrupolar and HO channels. We therefore compared the angular radiation pattern of the selected core-shell particle with an ideal MD reference.

Figure S4(a) shows the normalized far-field angular pattern of a pure magnetic dipole. Figure S4(b) shows the corresponding normalized angular patterns of the selected core-shell particle at, $\lambda_{\text{sel}}^{\text{PW},0} = 425.0$ nm for both the lossless candidate and the weakly lossy counterpart. The weakly lossy counterpart uses the same geometry and real refractive indices as the lossless candidate, with $k_{\text{core}} = 0, k_{\text{shell}} = 0.007143$. To quantify the difference between the selected-particle pattern and the pure-MD reference, we used the normalized root-mean-square deviation,

$$\text{RMS} = \left[\frac{1}{N}\sum_{i=1}^{N}(I_{\text{CS}}(\theta_i) - I_{\text{MD}}(\theta_i))^2\right]^{1/2},$$

where $I_{\text{CS}}(\theta_i)$ and $I_{\text{MD}}(\theta_i)$ are the normalized angular intensities of the core-shell particle and the pure MD reference, respectively. We also evaluated the Pearson correlation coefficient,

$$\rho = \frac{\sum_i [I_{\text{CS}}(\theta_i) - \bar{I}_{\text{CS}}]\,[I_{\text{MD}}(\theta_i) - \bar{I}_{\text{MD}}]}{\left[\sum_i (I_{\text{CS}}(\theta_i) - \bar{I}_{\text{CS}})^2\right]^{1/2}\left[\sum_i (I_{\text{MD}}(\theta_i) - \bar{I}_{\text{MD}})^2\right]^{1/2}}.$$

Here $\rho = 1$ would indicate identical angular-pattern shape after normalization, whereas larger RMS values indicate larger pattern mismatch. For the lossless selected particle, the comparison gives, $\text{RMS} = 0.630, \rho = 0.622$. For the weakly lossy

counterpart, the corresponding values are, $\mathrm{RMS} = 0.637, \rho = 0.604$. These values show that the selected core-shell particle does not reproduce the pure-MD angular pattern exactly, even though its low-order multipolar spectrum is strongly MD-favored. The small difference between the lossless and weakly lossy values also indicates that this pattern mismatch is not mainly caused by the weak dissipative perturbation. The origin of the mismatch is clarified by the modal-fraction analysis in Figure S4(d). At the selected wavelength, the ED, MD, EQ, and MQ channels are all small compared with the total HO background. Therefore, the low-order MD-favored condition identified in the main text does not translate into a fully pure far-field MD radiator. Instead, the plane-wave branch should be interpreted as an intrinsic optical benchmark: it identifies an anapole-assisted ED-suppressed, MD-favored low-order state, while also showing that full far-field purity is limited by residual HO radiation.

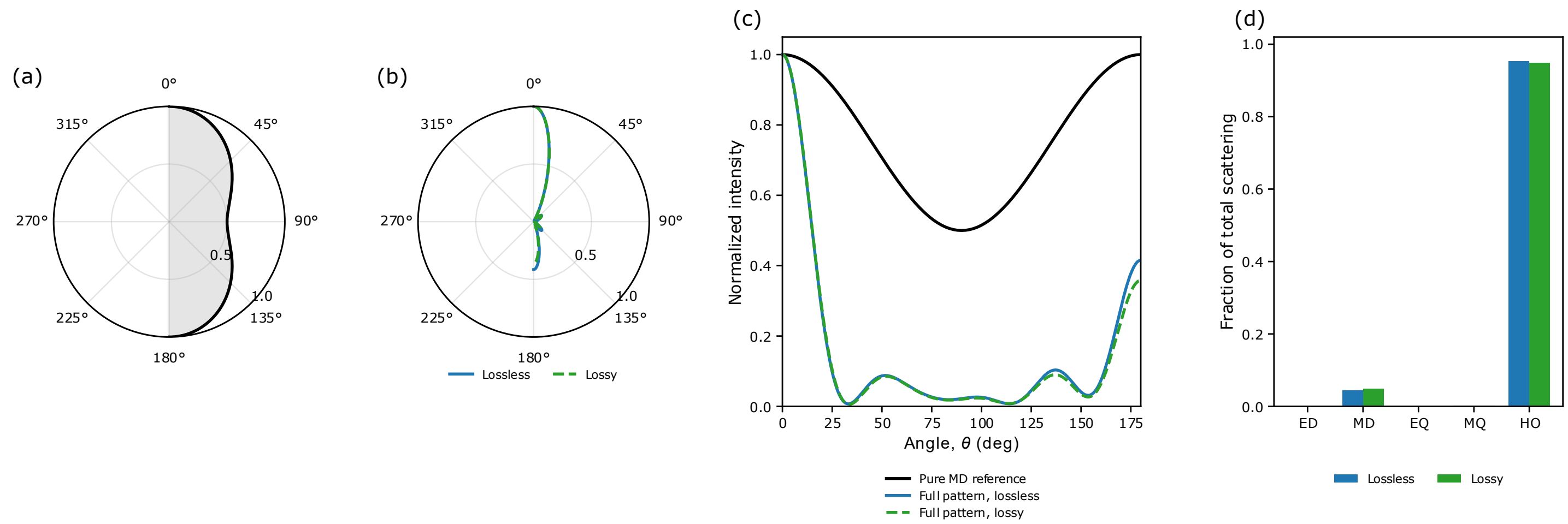

Figure S4. (a) Normalized angular radiation pattern of an ideal MD reference. (b) Normalized far-field angular patterns of the selected core-shell particle at $\lambda_{\mathrm{sel}}^{\mathrm{PW},0} = 425.0$ nm for the lossless case and for the weakly lossy counterpart with $k_{\mathrm{core}} = 0$ and $k_{\mathrm{shell}} = 0.007143$. (c) Pattern-difference comparison between the selected-particle far field and the pure-MD reference. The normalized RMS deviation and Pearson correlation coefficient quantify the mismatch. The lossless case gives $\mathrm{RMS} = 0.630$ and $\rho = 0.622$, while the weakly lossy case gives $\mathrm{RMS} = 0.637$ and $\rho = 0.604$. (d) Modal-fraction decomposition at the selected wavelength. Although the low-order spectrum is MD-favored, the total far-field pattern is still affected by HO radiation.

S5. Aloof-electron numerical implementation

The aloof-electron calculations were performed for a fast electron traveling outside the core-shell particle, with impact parameter

$$b > R.$$

The particle was embedded in air, and all media were treated as nonmagnetic. The electron velocity was expressed through the reduced velocity

$$\beta = \frac{v}{c},$$

with Lorentz factor

$$\gamma = \frac{1}{\sqrt{1-\beta^2}}.$$

For the 200 keV electron used as the representative beam condition in this work, $\beta \approx 0.695$. The aloof condition ensures that the electron excites the particle through its evanescent electromagnetic field without directly penetrating the core-shell structure.

The radiative response was analyzed through a beam-weighted multipolar CL decomposition [5,6]. For each wavelength, impact parameter, and electron velocity, the calculated CL signal was separated into ED, MD, EQ, MQ, and HO channels,

$$\Gamma_{\mathrm{CL}} = \Gamma_{\mathrm{ED}}^{\mathrm{CL}} + \Gamma_{\mathrm{MD}}^{\mathrm{CL}} + \Gamma_{\mathrm{EQ}}^{\mathrm{CL}} + \Gamma_{\mathrm{MQ}}^{\mathrm{CL}} + \Gamma_{\mathrm{HO}}^{\mathrm{CL}}.$$

The magnetic-dipole CL purity was then evaluated as

$$\eta_{\mathrm{MD}}^{\mathrm{CL}} = \frac{\Gamma_{\mathrm{MD}}^{\mathrm{CL}}}{\Gamma_{\mathrm{CL}}}.$$

This quantity measures the fraction of the radiative electron-excited signal carried by the MD channel.

In the numerical implementation, the electron trajectory enters through beam-weight factors that depend on the impact parameter b, velocity v, wavelength, and multipole order. These factors weight the electric- and magnetic-type spherical channels differently. Therefore, even for the same core-shell particle, the observed CL hierarchy can change when b/R or β is varied. This is the basis of the beam-conditioned selection discussed in the main text.

For compactness, the HO leakage was grouped as

$$\Gamma_{\mathrm{leak}}^{\mathrm{CL}} = \Gamma_{\mathrm{EQ}}^{\mathrm{CL}} + \Gamma_{\mathrm{MQ}}^{\mathrm{CL}} + \Gamma_{\mathrm{HO}}^{\mathrm{CL}}.$$

The selected aloof-electron state was identified by maximizing the CL selection score defined in Section S2, which rewards finite magnetic-dipole CL emission while penalizing ED and HO leakage. This criterion avoids selecting points where $\eta_{\mathrm{MD}}^{\mathrm{CL}}$ is high only because the total radiative signal is very weak.

The synthetic aloof-electron search was performed in three steps. First, the refractive-index pair was selected from an index-space search at fixed geometry and representative beam condition. Second, the geometry was optimized for the selected index pair. Third, the beam parameters β and b/R were scanned for the selected core-shell candidate. This procedure yielded the synthetic aloof benchmark reported in the main text, $n_{\mathrm{core}} = 4.90, n_{\mathrm{shell}} = 1.05$, $R = 230$ nm, $r/R = 0.375$, with selected wavelength $\lambda_{\mathrm{IMD}}^{\mathrm{CL}} = 875$ nm. At this operating point, the magnetic-dipole CL purity is

$$\eta_{\mathrm{MD}}^{\mathrm{CL}} \approx 0.795.$$

To verify that this state is not a boundary artifact, the selected candidate was further examined in an extended $(\lambda, b/R)$ operating-window map at fixed $\beta = 0.788$. The maximum in the extended map occurs near $b/R \approx 3.33$, close to the selected operating point, and gives nearly the same MD purity,

$$\eta_{\mathrm{MD}}^{\mathrm{CL}} \approx 0.798.$$

The final selected value $\beta = 0.788$ corresponds to a higher beam energy than the representative 200 keV condition used in the initial index and geometry searches; it is therefore best understood as the optimized beam-conditioned operating point within the scanned velocity range.

A line cut at $\lambda = 875$ nm was then used to compare the normalized total CL yield, normalized MD CL yield, selection score, and MD purity as a function of b/R. This line cut shows the expected yield–selectivity trade-off: increasing the aloof distance can preserve modal selectivity but reduces the absolute radiative yield.

Finally, weak material loss was introduced for the selected aloof candidate by scanning the extinction coefficients k_{core} and k_{shell} while keeping the geometry, real refractive indices, β, and b/R fixed. The best nonzero-loss point from this perturbative scan was

$$k_{\mathrm{core}} = 0, k_{\mathrm{shell}} = 0.005,$$

with

$$\eta_{\mathrm{MD}}^{\mathrm{CL}} \approx 0.793.$$

This confirms that the selected aloof-electron MD-favored state is not created by the strictly lossless approximation and is not immediately destroyed by a small dissipative perturbation.

No separate Cartesian *P–T* reconstruction was used as the defining diagnostic for the aloof-optimized state. The reason is that the aloof-electron analysis addresses a beam-weighted radiative readout rather than a new current-level anapole condition. Therefore, the relevant observable is the CL multipolar hierarchy,

$$\Gamma_{\mathrm{MD}}^{\mathrm{CL}}, \Gamma_{\mathrm{ED}}^{\mathrm{CL}}, \Gamma_{\mathrm{leak}}^{\mathrm{CL}},$$

while the Cartesian *P–T* diagnostic is used only for the intrinsic plane-wave benchmark. The implementation used here is the semi-analytical in-house Python approach described in Section S1.1; no spatial meshing or commercial electromagnetic solver was used. Penetrating trajectories are not included in the present implementation; recent analytical developments for spherical core-shell nanoparticles under aloof and penetrating fast-electron trajectories show that this regime requires a separate treatment [7].

S5.1. Angular balance of the selected aloof-electron CL states

To address the angular directionality of the selected cathodoluminescence states, we evaluated the φ-averaged angular emission profiles for the synthetic aloof-electron benchmark and for the real-material Si@MgF_2 candidate. The forward and backward hemispheres are defined relative to the electron propagation axis, $+z$. The forward component, F, was obtained by integrating the φ-averaged angular intensity over 0-90°, while the backward component, B, was obtained over 90-180°. In both cases, the integration included the solid-angle weighting factor $sin(\theta)$. The intrinsic angular-balance parameter was then defined as $A_{FB} = (F - B)/(F + B)$, where positive A_{FB} indicates preferential emission into the forward hemisphere.

Figure S5 shows the resulting angular balance. For the synthetic aloof-electron benchmark at $\lambda = 875$ nm and $b/R = 3.0$, the hemispherical fractions are $F = 0.579$ and $B = 0.421$, giving $A_{FB} = 0.157$. This indicates a modest forward bias rather than a

strongly unidirectional radiation pattern. For the real-material Si@MgF_2 candidate at λ = 895 nm and b/R = 2.5, the angular imbalance is stronger, with F = 0.692 and B = 0.308, giving A_{FB} = 0.383. Thus, the real-material candidate is preferentially forward-emitting, although a non-negligible backward component remains.

This directionality has practical implications for substrate-supported CL experiments. If the nanoparticle is placed on a substrate, the forward-directed component can partly couple into the substrate and may be reduced in an air-side collection geometry. At the same time, the finite backward component remains directly accessible to air-side collection. Depending on the substrate reflectivity, thickness, and collection geometry, part of the forward-directed radiation may also be indirectly recovered through reflection. The present analysis gives the intrinsic free-space angular balance of the selected CL states; a full substrate-resolved, angle-resolved collection model including finite numerical aperture and substrate optical constants is outside the scope of this work.

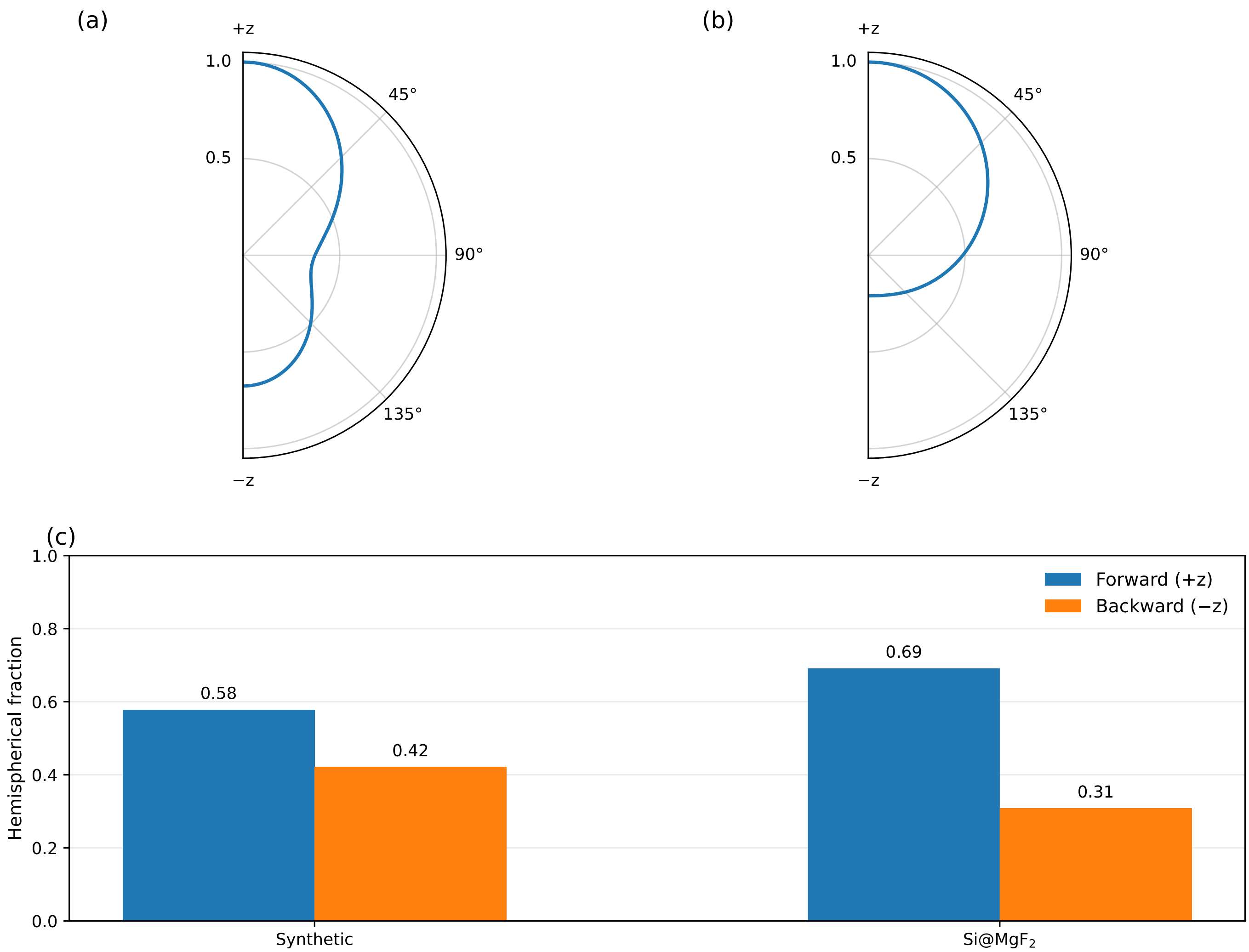

Figure S5. (a) φ-averaged normalized angular profile for the synthetic aloof-electron benchmark at λ = 875 nm and b/R = 3.0. (b) φ-averaged normalized angular profile for the real-material Si@MgF_2 candidate at λ = 895 nm and b/R = 2.5. Forward and backward directions are defined relative to the electron propagation axis, $+z$. The intrinsic angular-balance parameter is defined as $A_{FB} = (F - B)/(F + B)$, where F and B are obtained from hemispheric integration of $I(\theta)sin(\theta)$ over 0-90° and 90-180°, respectively. The synthetic case gives F = 0.579, B = 0.421, and A_{FB} = 0.157, indicating a modest forward bias. The Si@MgF_2 case gives F = 0.692, B = 0.308, and A_{FB} = 0.383, indicating a stronger forward bias. (c) Corresponding forward and backward hemispherical fractions for the two selected CL states.

S6. Real-material candidate

The synthetic aloof-electron analysis was used to isolate the beam-conditioned selection physics without tying the result to a specific dispersive material system. To test whether the same design tendency can survive in a realistic dielectric pair, we considered a compact Si@MgF_2 core-shell particle as a representative real-material candidate. This calculation was not intended as an exhaustive materials optimization, but as a proof-of-principle translation of the synthetic aloof-electron design rule. The core was modeled as silicon and the shell as magnesium fluoride. The optical constants were treated as wavelength-dependent quantities,

$$m_{\mathrm{Si}}(\lambda) = n_{\mathrm{Si}}(\lambda) + ik_{\mathrm{Si}}(\lambda),$$
$$m_{\mathrm{MgF_2}}(\lambda) = n_{\mathrm{MgF_2}}(\lambda) + ik_{\mathrm{MgF_2}}(\lambda).$$

For numerical calculations, the silicon core was described by a representative dispersive dielectric model with weak absorption in the near-infrared spectral window used here, while the MgF_2 shell was treated as a low-index, low-loss dielectric. The same aloof-electron CL decomposition and selection procedure described in Sections S2 and S5 were then applied to this real-material particle. The selected real-material geometry was, $r = 100$ nm, $R = 120$ nm, corresponding to a shell thickness of $t = R - r = 20$ nm. The aloof-electron impact parameter was, $b/R = 2.5$. The selected operating wavelength was, $\lambda_{\mathrm{sel}} = 895$ nm. At this wavelength, the CL modal fractions were, $\frac{\Gamma_{\mathrm{ED}}^{\mathrm{CL}}}{\Gamma_{\mathrm{CL}}} = 0.260$, $\frac{\Gamma_{\mathrm{MD}}^{\mathrm{CL}}}{\Gamma_{\mathrm{CL}}} = 0.729$, and $\frac{\Gamma_{\mathrm{leak}}^{\mathrm{CL}}}{\Gamma_{\mathrm{CL}}} = 0.011$, where $\Gamma_{\mathrm{leak}}^{\mathrm{CL}} = \Gamma_{\mathrm{EQ}}^{\mathrm{CL}} + \Gamma_{\mathrm{MQ}}^{\mathrm{CL}} + \Gamma_{\mathrm{HO}}^{\mathrm{CL}}$. Thus, the real-material Si@MgF_2 particle retains a magnetic-dipole-favored CL hierarchy: the MD contribution is clearly larger than the ED contribution, while quadrupolar and HO leakage remains small. Compared with the synthetic aloof-electron benchmark, the real-material response is less selective, but it preserves the same qualitative design rule.

Table S2. Parameters and modal fractions of the representative real-material Si@MgF_2 core-shell candidate used in Figure 5. The modal fractions are evaluated at $\lambda_{\mathrm{sel}} = 895$ nm under aloof-electron excitation with $b/R = 2.5$. The result shows that the real-material candidate preserves a MD-favored CL response, although with reduced selectivity compared with the synthetic benchmark.

Parameter	Value
Core material	Si [8]
Shell material	MgF_2 [9]
Core radius, (r)	100 nm
Outer radius, (R)	120 nm
Shell thickness, (t)	20 nm
Impact parameter	$b/R = 2.5$
Selected wavelength	$\lambda_{\mathrm{sel}} = 895$ nm
ED fraction	0.260
MD fraction	0.729
Leakage fraction, EQ + MQ + HO	0.011

Table S3 summarizes the same real-material candidate together with the synthetic aloof-electron benchmark to clarify how much MD selectivity is retained when moving from the high-index synthetic optimum to the practical Si@MgF_2 system.

Table S3. Comparison between the synthetic aloof-electron benchmark and the real-material Si@MgF_2 candidate.

Case	Material / index regime	Geometry	Wavelength	ED fraction	MD fraction	Leakage
Synthetic aloof benchmark	n_{core} = 4.90, n_{shell} = 1.05	R = 230 nm, r/R = 0.375	875 nm	0.112	0.795	0.093
Real-material candidate	Si@MgF_2	r = 100 nm, R = 120 nm	895 nm	0.260	0.729	0.011

The comparison shows that the real-material Si@MgF_2 candidate retains a magnetic-dipole-favored CL hierarchy, although the ED fraction increases compared with the synthetic benchmark. Therefore, the high-index synthetic optimum should be interpreted as a limiting design case rather than as a strict material requirement.

References

[1] Aden, A. L.; Kerker, M. Scattering of Electromagnetic Waves from Two Concentric Spheres. *J. Appl. Phys.* **1951**, *22*, 1242–1246.

[2] Radescu, E. E.; Vaman, G. Exact Calculation of the Angular Momentum Loss, Recoil Force, and Radiation Intensity for an Arbitrary Source in Terms of Electric, Magnetic and Toroid Multipoles. *Phys. Rev. E* **2002**, *65*, 046609.

[3] Miroshnichenko, A. E.; Evlyukhin, A. B.; Yu, Y. F.; Bakker, R. M.; Chipouline, A.; Kuznetsov, A. I.; Luk'yanchuk, B.; Chichkov, B. N.; Kivshar, Y. S. Nonradiating Anapole Modes in Dielectric Nanoparticles. *Nat. Commun.* **2015**, *6*, 8069.

[4] Stamatopoulou, P. E.; Maciel-Escudero, C.; Mortensen, N. A.; Rockstuhl, C.; Tserkezis, C. Analytic Methods in Electron Energy-Loss and Cathodoluminescence Spectroscopy of Planar and Spherical Nanostructures: Tutorial. *J. Opt. Soc. Am. B* **2025**, *42* (7), 1620.

[5] García de Abajo, F. J. Optical Excitations in Electron Microscopy. *Rev. Mod. Phys.* **2010**, *82*, 209–275.

[6] García de Abajo, F. J. et al. Roadmap for Quantum Nanophotonics with Free Electrons. *ACS Photonics* **2025**, 12, 9, 4760–4817.

[7] Brandt, A.; Tserkezis, C.; Rockstuhl, C.; Elli Stamatopoulou, P. Probing Strong Coupling in Core-Shell Nanoparticles with Fast Electron Beams. *arXiv [physics.optics]*, 2026. https://doi.org/10.48550/arXiv.2603.13797.

[7] Green, M. A. Self-Consistent Optical Parameters of Intrinsic Silicon at 300 K Including Temperature Coefficients. *Sol. Energy Mater. Sol. Cells* **2008**, *92*, 1305–1310.

[8] Dodge, M. J. Refractive Properties of Magnesium Fluoride. *Appl. Opt.* **1984**, *23*, 1980–1985.
